# Identification and validation of ferroptosis related markers in erythrocyte differentiation of umbilical cord blood-derived CD34^+^ cell by bioinformatic analysis

**DOI:** 10.3389/fgene.2024.1365232

**Published:** 2024-07-30

**Authors:** Qian Liu, Ze Lin, Minghui Yue, Jianbo Wu, Lei Li, Daqi Huang, Yipeng Fang, Xin Zhang, Tao Hao

**Affiliations:** ^1^ Department of Cardiology, Binzhou Medical University Hospital, Binzhou, Shandong, China; ^2^ Shantou University Medical College, Shantou, Guangdong, China; ^3^ Department of Critical Care Medicine, Tianjin Medical University General Hospital, Tianjin, China; ^4^ Laboratory of Molecular Cardiology, The First Affiliated Hospital of Shantou University Medical College, Shantou, Guangdong, China; ^5^ Laboratory of Medical Molecular Imaging, The First Affiliated Hospital of Shantou University Medical College, Shantou, Guangdong, China; ^6^ Department of Colorectal Surgery, Binzhou Medical University Hospital, Binzhou, Shandong, China

**Keywords:** ferroptosis, erythrocyte differentiation, human umbilical cord blood-derived CD34^+^ cell, immunocyte infiltration, bioinformatic analysis

## Abstract

Ferroptosis has been observed to play an important role during erythrocyte differentiation (ED). However, the biological gene markers and ferroptosis mechanisms in ED remain unknown. We downloaded the datasets of ED in human umbilical cord blood-derived CD34^+^ cells from the Gene Expression Omnibus database. Using median differentiation time, the sample was categorized into long and short groups. The differentially expressed ferroptosis-related genes (DE-FRGs) were screened using differential expression analysis. The enrichment analyses and a protein–protein interaction (PPI) network were conducted. To predict the ED stage, a logistic regression model was constructed using the least absolute shrinkage and selection operator (LASSO). Overall, 22 DE-FRGs were identified. Ferroptosis-related pathways were enriched using Gene Ontology and the Kyoto Encyclopedia of Genes and Genomes. Gene Set Enrichment Analysis and Gene Set Variation Analysis revealed the primary involvement of DE-FRGs in JAK-STAT, MAPK, PI3K-AKT-mTORC1, WNT, and NOTCH signaling pathways. Ten-hub DE-FRGs were obtained using PPI analysis. Furthermore, we constructed mRNA-microRNA (miRNA) and mRNA-transcription factor networks. Immune cell infiltration levels differed significantly during ED. LASSO regression analysis established a signature using six DE-FRGs (*ATF3, CDH2, CHAC1, DDR2, DPP4,* and *GDF15*) related to the ED stage. Bioinformatic analyses identified ferroptosis-associated genes during ED, which were further validated. Overall, we identified ferroptosis-related genes to predict their correlations in ED. Exploring the underlying mechanisms of ferroptosis may help us better understand pathophysiological changes in ED and provide new evidence for clinical transformation.

## 1 Introduction

Erythropoiesis is a complex, tightly regulated process. Altered erythroid production leads to various types of anemia and hemoglobin disorders including thalassemia syndrome, inherited bone marrow failure, chronic anemia, polycythemia vera, and sickle cell anemia (Zhang Y. et al., 2022). Billions of people are affected by various erythrocyte-related diseases worldwide, posing a significant public health challenge (Srivastava et al., 2022). Erythrocyte transfusion is indispensable and irreplaceable not only for patients with anemia and hemoglobin disorders, but also for modern medical practices, such as cancer treatment and surgery (Lee et al., 2023). However, the whole process, from blood collection to clinical use, consumes a significant amount of resources, and there are many problems to solve, such as the spread of pathogenic microorganisms, the cost of the whole process, and approximately 118.5 million units of blood collected globally each year, which are insufficient to meet the annual global demand of over 300 million units for blood transfusions (Christaki et al., 2019). An aging population and emerging risks of viruses and pathogens exacerbate the problem of supply shortages. The development of alternative blood transfusion products helps mitigate erythrocyte shortages and transfusion limitations. Each unit of transfused blood contains a trillion erythrocytes. However, obtaining such large quantities of erythrocytes *in vitro* involve overcoming several challenges. The bottleneck of low adult β-hemoglobin expression and nucleation efficiency remains to be resolved. The regulatory mechanism during erythroid differentiation will help to further study and ultimately identify the molecular pathways involved in the pathological process of erythroid diseases and provide great promise for transfusion medicine and novel cell-based therapies.

Developing erythroid precursors absorb exceptionally large amounts of iron to accommodate increased synthesis of heme (Ryu et al., 2017; Philpott, 2020). Iron could be released from ferritin, where most of the cellular iron is stored through a process known as “ferritinophagy” (Dowdle et al., 2014; Mancias et al., 2014). Ferritinophagy is an important step for iron release before mitochondrial iron import and heme biosynthesis (Nilsson et al., 2009; Mancias et al., 2015). Ferritinophagy initiates ferroptosis by promoting iron accumulation. Ferroptosis is an active mode of cell death, defined as catalytic Fe(II)-dependent regulated necrosis accompanied by lipid peroxidation (Toyokuni et al., 2023). Ferroptosis involves embryonic hematopoiesis, particularly erythropoiesis in rats (Zheng et al., 2021). Ferroptosis is observed in the extraembryonic endodermal component of the visceral yolk sac, which induces blood precursors, and in embryonally nucleated erythrocytes that, disappear in enucleated erythrocytes (Zheng et al., 2021). Ferroptosis inhibitors significantly delay erythrocyte enucleation. Additionally, ferroptosis may mediate B-cells differentiation (Chen et al., 2022). Abnormal ferroptosis damages the development erythrocytes, leading to erythropoiesis suppression and anemia (Chen et al., 2022). Ferroptosis has new therapeutic potential for blood cell-related diseases. In many patients with heart failure, inflammation, and oxidative stress lead to an iron-deficient state, which can limit erythropoiesis in erythroid precursors (Packer, 2022). Increasing the levels of cytosolic Fe (2+) available to the mitochondria enables the synthesis of heme from erythroid precursors (Packer, 2022). However, the genes significantly related to ferroptosis in erythropoiesis are unknown, and whether ferroptosis genes can be used as biomarkers to discriminate between early and late differentiation is yet to been reported.

CD34^+^ cells isolated from umbilical cord blood (UCB) serve as a valuable model system to study gene regulation of erythropoiesis. This study obtained data on the erythrocyte differentiation (ED) of human umbilical cord blood-derived CD34^+^ cells from the Gene Expression Omnibus (GEO) database for bioinformatic analysis to screeng differentially expressed genes (DEGs) between early and late differentiation. Subsequently, the differentially expressed ferroptosis-related genes (DE-FRGs) were acquired. Their ability to diagnose the differentiation stage, biological functions, and regulatory networks was analyzed. We also identified hub genes and predicted their association with immune cell infiltration. Our findings explain the potential role of ferroptosis in erythropoiesis and may provide new directions for treating of erythrocyte-related diseases.

## 2 Methods

### 2.1 Data collection

Expression profile data associated with ED were obtained from the GEO database (Barrett et al., 2013). ED-related datasets were retrieved using the following keywords: “umbilical cord blood CD34^+^ cells” and “*Homo sapiens*.” Our study comprised data from 46 data from UCB-CD34^+^ cell-derived erythroid progenitors at different time points of differentiation. We downloaded the (ED)-related datasets GSE49438 (a total of 12 samples harvested on day 21, 42, 49, and 56) (Li B. et al., 2014), GSE118537 (a total of 28 samples harvested on day 0, 2, 4, 6, 7.5, 8, 8.5, 10, 10.5, 11, 11.5, 12, 14, and 16) (Gillespie et al., 2020) and GSE156306 (a total of 6 samples harvested on day 8, 10, and 12) (Guo et al., 2020) using the R package GEOquery (Davis and Meltzer, 2007). The details of the database are presented in Table 1.

**TABLE 1:** List of dataset information.

Items	GSE49438	GSE118537	GSE156306
Platform	GPL10558	GPL11154	GPL20301
Species	*Homo sapiens*	*Homo sapiens*	*Homo sapiens*
Disease	erythrocyte differentiation	erythrocyte differentiation	erythrocyte differentiation
Tissue	umbilical cord blood	umbilical cord blood	umbilical cord blood
Samples in Case group	12	28	6
References	Li et al. (2014a)	Gillespie et al. (2020)	Guo et al. (2020)

Additionally, 753 ferroptosis-related genes (FRGs) were obtained by combining and deduplicating genes from the GeneCards (Stelzer et al., 2016), Molecular Signatures Database (MSigDB) (Liberzon et al., 2015) and FerrDb online databases (Zhou and Bao, 2020).

### 2.2 Identification of DE-FRGs

We used R package sva (Leek et al., 2012) to de-batch the three datasets and obtained the integrated GEO dataset as an ED-dataset (Supplementary Figure S1). ED can be categorized into the early proliferation and late differentiation stages. Based on the median cultivation time (11 days) of the ED dataset, we divided the group into long cultivation time (long) and short cultivation time (short) groups for difference analysis. According to the three literatures, most cells in the short and long groups were in the proliferation and differentiation stages, respectively. The linear model for microarray data (limma) package was used to identify DEGs between samples from long group and those from those from short group. The *p*-value correction method used was BH procedure. The screening thresholds for DEGs are |log2FoldChange (logFC)| >1.5 and adjusted *p* < 0.05 and presented as a volcano map drawn using the R package ggplot2. The upregulated DEGswith log2FC > 1.5 and adjusted *p* < 0.05 and the downregulated DEGs as genes with log2FC < −1.5 and adjusted *p* < 0.05 were intersected with FRGs and presented as Venn diagram, respectively. The DEG results were obtained using volcano plots and heat maps drawn with the R package ggplot2 and pheatmap, respectively. DE-FRGs were identified by intersecting the DEGs and FRGs. We used the R package RCircos (version 1.2.2) (Zhang et al., 2013) to draw a chromosome localization map and observe the distribution positions of the screened DE-FRGs on human chromosomes. The DE-FRG expression in the ED dataset was displayed as a heatmap drawn using the R package pheatmap.

### 2.3 Gene ontology (GO) and Kyoto encyclopedia of genes and genomes (KEGG) enrichment analysis

GO and KEGG enrichment analyses were conducted using the R package clusterProfiler (Version 4.10.0) and R package GOplot (Version: 1.0.2), respectively (Yu et al., 2012). The threshold was set as *p* < 0.05, the false discovery rate (FDR) value (q.Value) was <0.25. Combined with the log2FC values, enrichment analyses were performed by calculating Z-scores using the R package GOplot.20.

### 2.4 GSEA

We retrieved the gene set “c2.cp.v7.2. symbols” in theMSigDB. We conducted enrichment analysis on all genes within the long and short groups of the ED-dataset using the R package cluster Profiler. The parameters were as follows: the seed was 2020, the number of calculations was 10,000, and each gene set contained at least five genes, and at most 500 genes, and the screening criteria for significant enrichment were *p* < 0.05 and FDR value (q.Value) < 0.25.

### 2.5 Gene set variation analysis (GSVA)

We obtained the gene set “H.A.v7.4. Symbols.gmt” from the MSigDB database for GSVA and investigated the variation in biological processes between the long and short groups of the ED dataset. Statistical significance for enrichment was set at *p* < 0.05.

### 2.6 PPI network, mRNA-miRNA, and mRNA--transcription factor (TF) prediction networks

The STRING database was used to construct a PPI network associated with DE-FRGs (with a minimum required interaction score of 0.400). The PPI network model was visualized using the Cytoscape software (version 3.9.1). The GeneMANIA website was used to predict the functional similarity of the selected DE-FRGs and construct an interaction network. ENCORI and CHIPBase databases (Li J. H. et al., 2014) (version 3.0) (https://rna.sysu.edu.cn/chipbase/database) predicted that the miRNAs supported more than three databases and TF supported more than 6 document interaction with DE-FRGs, respectively. Subsequently, the Cytoscape software was used to visualize the interaction between mRNA-miRNAs and mRNA-TF.

### 2.7 Construction of subtypes of erythrocyte differentiation though consistent clustering

Cluster analysis based on DE-FRGs was performed using Consensus Cluster Plus (Wilkerson and Hayes, 2010) with the standard (the number of clusters was set between two and eight, and 80% of the total samples were extracted by repeating 1,000 times = “km,” distance = “euclidean”).

### 2.8 Immune infiltration analysis of single sample gene set enrichment analysis (ssGSEA) ssGSEA algorithm and CIBERSORT algorithm

Based on the expression of FRGs in the dataset, the FRGs score of each sample was obtained using the ssGSEA algorithm to represent the FRGs expression level of the sample. The group was then divided into high and low FRGs scores bounded by the median FRGs score.

Based on the median FRGs of the samples, the gene set was divided into high and low FRG score groups for the gene correlation analysis. The difference in infiltration abundance of immune cells between groups with high and low FRG scores in the ED dataset of 28 immune cell types is demonstrated using a boxplot plot, and the correlation between immune cells and FRGs is shown through correlation heat map and correlation scatter plot.

CIBERSORTx is a deconvolution of the transcriptome expression matrix that is used to estimate the composition and abundance of immune cells among mixed cells. The gene expression matrix data were grouped and uploaded to the CIBERSORTx website. Combined with the LM22 characteristic gene matrix, immune cell infiltration matrix data were obtained and displayed in stacked bar charts. Subsequently, we combined the gene expression matrix to calculate the correlation between immune cells and DE-FRGs and drew a correlation heat map using the R package pheatmap. The correlation between immune cells and FRGs was determined using Spearman’s rank correlation coefficient and visualized using the R package ggplot2.

### 2.9 The least absolute shrinkage and selection operator (LASSO) model, and logistic model identification of optimal diagnostic genes

To obtain an ED diagnostic model for FRGs, we used 10x cross-validation on the ED-dataset with a seed number of 123, performed LASSO regression, and ran 1,000 cycles to prevent overfitting (Zhang et al., 2023). Subsequently, we analyzed the DE-FRGs in the LASSO diagnostic model using a univariate logistic model. DE-FRGs meeting the threshold (*p* < 0.10) were identified using a multivariate logistic model. A nomogram was drawn using RMS to show the relationship between DE-FRGs in the diagnostic model based on the results of the multi-factor logistic model. Finally, we drew a calibration curve using calibration analysis to evaluate the accuracy and resolution of the logistic model based on the DE-FRGs.

### 2.10 RNA isolation and quantitative reverse-transcription PCR (RT-qPCR)

One million CD34^+^ cells from 100 mL of human umbilical cord blood were purified using anti-CD34 antibodies linked to magnetic microbeads. Subsequently, primary human CD34^+^ hematopoietic progenitor cells were differentiated *ex vivo* in IMDM (Thermo Fisher Scientific, Waltham, Massachusetts) containing 20% knockout serum replacement and 2 mM GlutaMAX (Thermo Fisher Scientific), 25 μg/mL insulin, 150 μg/mL transferrin, 40 μg/mL inositol, 10 μg/mL folic acid, 90 ng/mL ferric nitrate, 900 ng/mL ferrous sulfate, and 160 μM monothioglycerol (all from Sigma-Aldrich, St. Louis, Missouri). From induction day 0 to day 8, the cells were cultured in medium supplemented with 1 nM dexamethasone (Sigma-Aldrich), 10 ng/mL IL-3,100 ng/mL stem cell factor (both from Peprotech, Rocky Hill, New Jersey), and 3 U/mL erythropoietin (Epo, R&D Systems, Minneapolis, Minnesota). From induction day 8–14, expanded erythroblasts were cultured in the presence of 3 U/mL Epo and 100 ng/mL stem cell factor. From day 14, the erythroblasts were cultured in the presence of 3 U/mL Epo.

From days 8–12, the major population of the cells was in the proliferation stage, while from day 16, the major population of the cells was in the differentiation stage. Cells were separately harvested after 8, 12, and 16 days of differentiation for RNA isolation and qRT-PCR analysis. RNA extraction, cDNA synthesis, and qPCR were performed as previously described (Liu et al., 2020). The comparing relative fold expression differences of each gene were normalized to the expression of day 8 by the method of 2^−ΔΔCt^. The primer sequences are listed in Supplementary Table S1.

### 2.11 Statistical analysis

All data processing and statistical analyses were performed using the R software Version 4.1.2 (The R Foundation for Statistical Computing, Vienna, Austria). Continuous variables were compared using Wilcoxon rank-sum test. One-way analysis of variance followed by Dunnett’s multiple comparison test was used to compare means among the different groups. A ROC curve was generated to assess the model performance. Correlations were calculated using Spearman correlation analysis. A two-sided *p* < 0.05 was considered statistically significant.

## 3 Results

### 3.1 Identification of DEGs and ferroptosis-related DEGs

**FIGURE 1:**
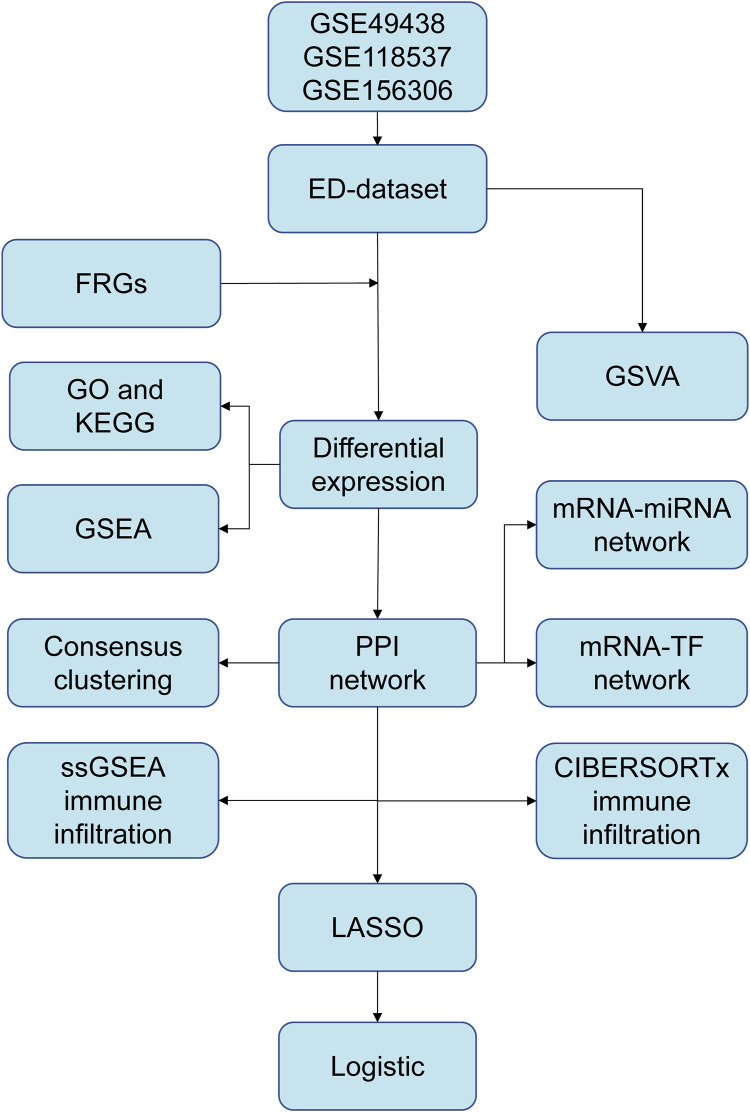
The flow chart of data preparation, processing, and analysis. ED, erythrocyte differentiation. FRGs, ferroptosis-related genes. GO, Gene Ontology. KEGG, Kyoto Encyclopedia of Genes and Genomes. GSVA, Gene Set Variation Analysis. GSEA, Gene Set Enrichment Analysis. PPI, Protein-protein interaction. TF, transcription factor. ssGSEA, single-sample gene set enrichment analysis. LASSO, least absolute shrinkage and selection operator.

**FIGURE 2:**
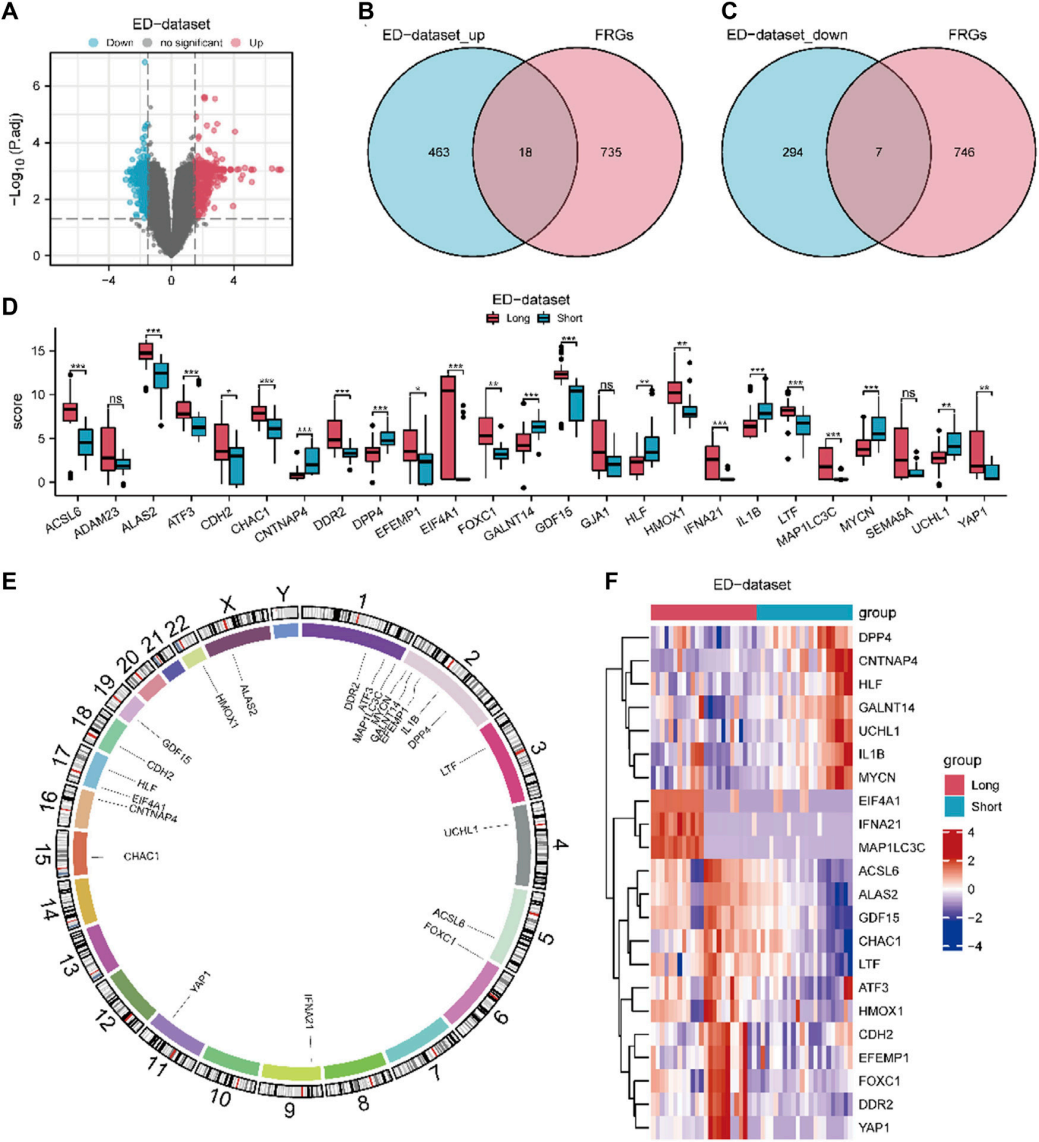
Expression differences of FRGs in ED datasets. **(A)** Volcano map of DEGs between long and short group. The DE-FRGs were obtained after intersecting the up-regulated **(B)** and down-regulated **(C)** DEGs with FRGs. **(D)** Grouping comparison diagram of DE-FRGs in ED-dataset. **(E)** Chromosome mapping of DE-FRGs. **(F)** Heat map of 22 DE-FRGs in ED-dataset. The comparison between two grous is performed using the Wilcoxon rank sum test. The comparison is performed using the Wilcoxon rank sum test. *P adj < 0.05, and **P adj< 0.01, ***P adj < 0.001, DEGs, differentially expressed genes. FRGs, ferroptosis-related genes. DE-FRGs, differentially expressed ferroptosis related to genes. ED, erythrocyte differentiation.

The workflow chart of this study is shown in Figure 1. A differential study of long and short samples revealed 782 DEGs, of which 481 and 301 were upregulated and down-regulated in long samples, respectively (Figure 2A). The intersections of DEGs and FRGs yielded 25 DE-FRGs, of which 18 and 7 were up-regulated (Figure 2B) and downregulated in long samples (Figure 2C), respectively. The grouping comparison diagram (Figure 2D) shows that 22 of the 25 DE-FRGs were statistically significant (*p* < 0.05). The positions of these 22 DE-FRGs on the human chromosomes were used to draw a chromosomal localization map (Figure 2E). These 22 FRGs were mainly distributed on chromosomes 1 and 2. ATF3, DDR2 and MAP1LC3C are located on chromosome 1, DPP4, EFEMP1, GALNT14, IL1B and MYCN are located on chromosome 2, and there are only 0–2 FRGs distributed on other chromosomes.

A heat map (Figure 2F) shows the expression of the 22 DE-FRGs in ED-dataset. To determine the diagnostic value of discriminating the early stage from the late stage of ED, ROC curves of the 22 FRGs were plotted (Supplementary Figure S2). Except for CDH2 (area under the curve [AUC] = 0.674), all other 21 FRGs (AUC 0.7–0.9) demonstrated moderate predictive accuracy.

### 3.2 Enrichment analysis reveals crucial processes and pathways during ED

**FIGURE 3:**
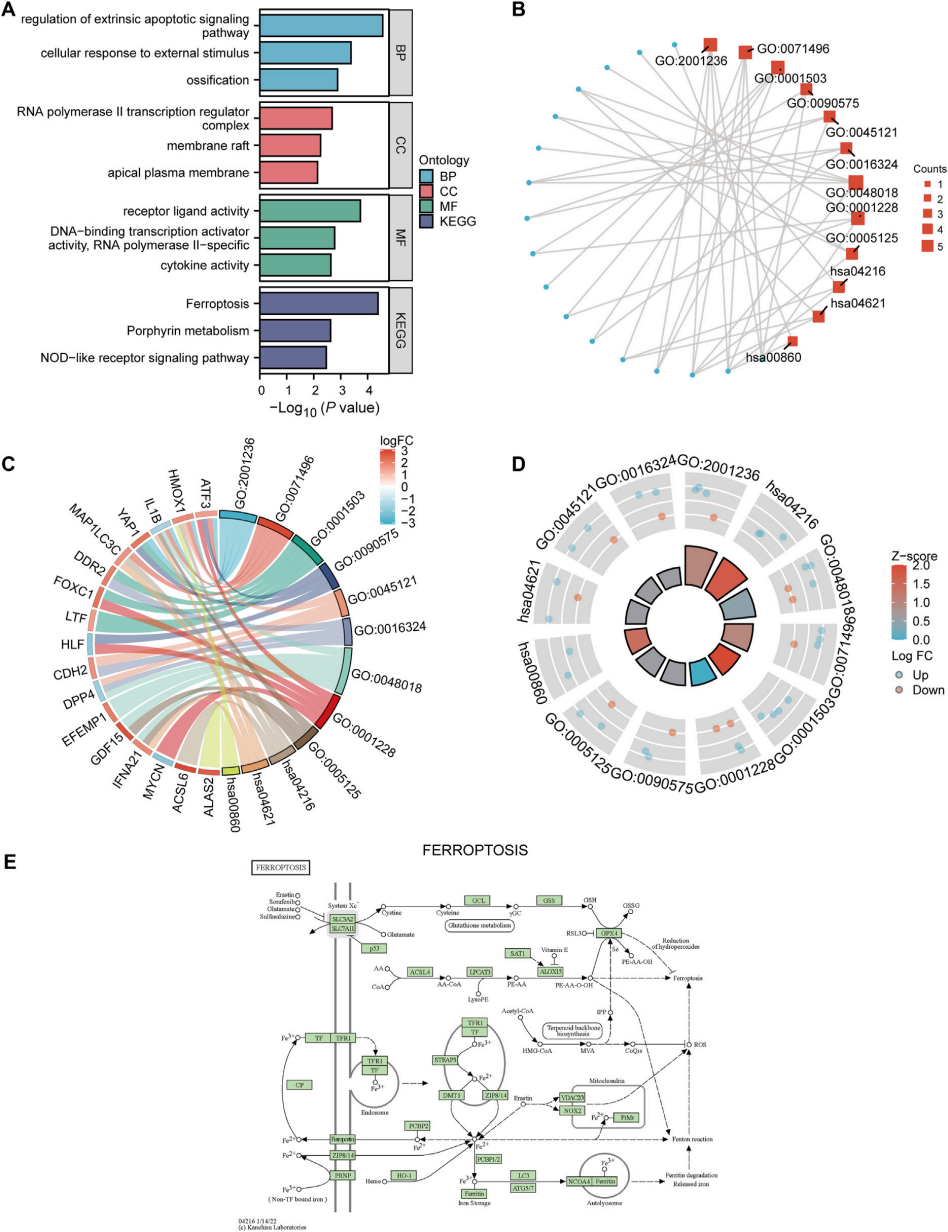
GO and KEGG analysis. **(A)** Bar graph showing the results of GO and KEGG analysis. **(B)** GO enrichment analysis results of DE-FRGs are shown in a circular network diagram. DE-FRGs combined log2FC are shown in the chord diagram **(C)** and donut plot **(D)**. The red and blue dots represent up-regulated (log2FC > 0) and t down-regulated (log2FC < 0) genes, respectively. **(E)** KEGG pathway diagram of ferroptosis (hsa04216). DE-FRGs, differentially expressed ferroptosis-related genes. GO, Gene Ontology. KEGG, Kyoto Encyclopedia of Genes and Genomes.

To elucidate the biological functions and pathways that were associated with the 22 DE‐FRGs, we then performed GO and KEGG analysis analyses, which are demonstrated by using the bar map (Figure 3A) and circular network diagram (Figure 3B), respectively. The GO analysis suggested that these DE‐FRGs were mainly enriched in the regulation of extrinsic apoptotic signaling pathway, RNA polymerase II transcription regulator complex, and receptor receptor-ligand activity. Moreover, the results of KEGG revealed that these DE‐FRGs were mainly enriched in the ferroptosis, and NOD-like receptor signaling pathways. Subsequently, a chord diagram (Figure 3C) and donut plot (Figure 3D) were constructed to show that these 22 DE-FRGs underwent GO and KEGG enrichment analysis of the combined log2FC. The results showed that ossification (GO:0001503) was significantly up-regulated. We selected a representative KEGG pathway to construct a ferroptosis pathway diagram (Figure 3E).

**FIGURE 4:**
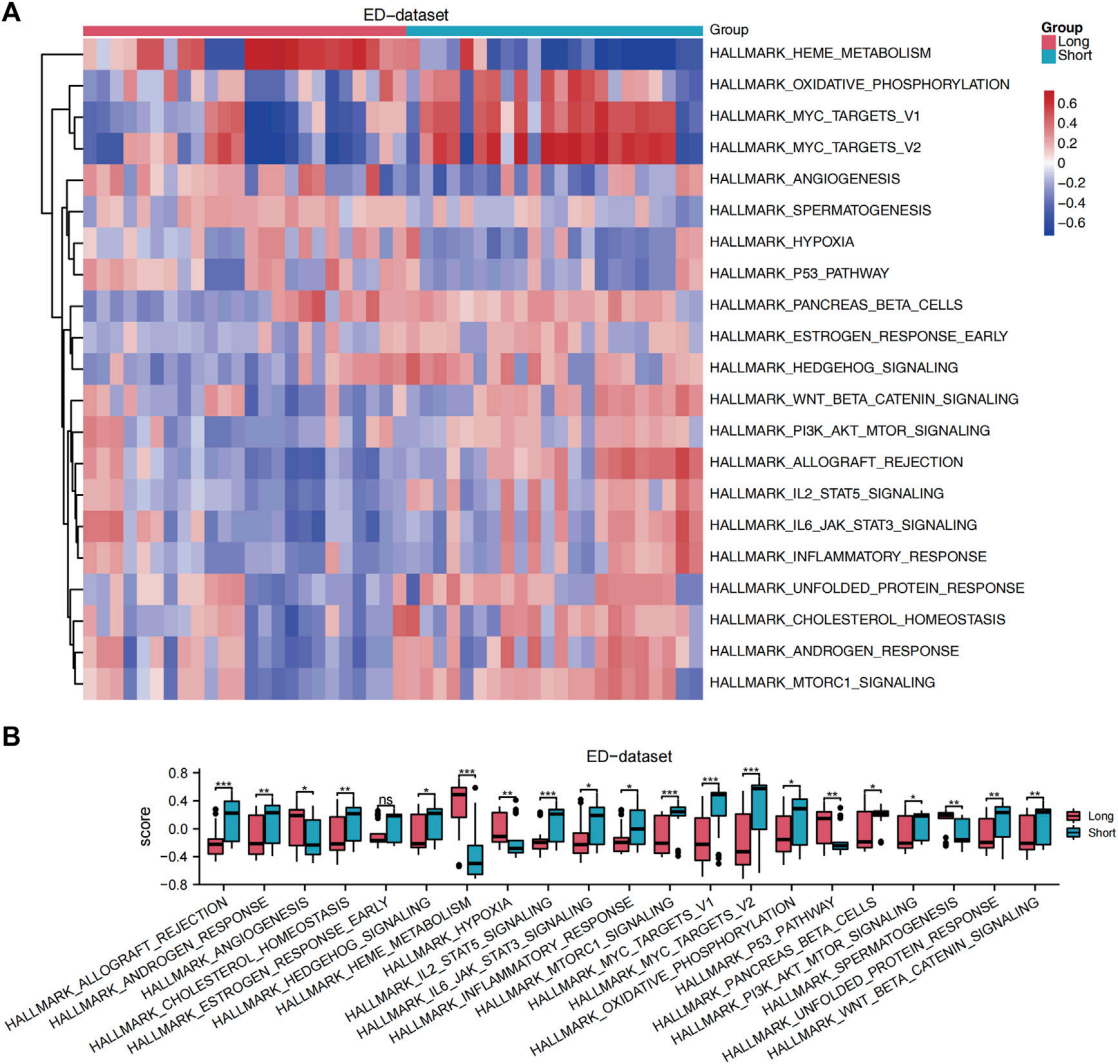
GSVA. **(A)** A heat map showed the scores of gene set functions in GSVA. **(B)** GSVA of enrichment pathways with statistical differences between the long and short groups. The comparison is performed using the Wilcoxon rank sum test. *p < 0.05, **p < 0.01, ***p < 0.001, ED, erythrocyte differentiation. GSVA, Gene Set Variation Analysis.

GSEA was performed to analyze the relationship between all gene expression and participating biological processes, affected cell components, and molecular functions of ED-datasets. GSEA indicated that the 22 DE-FRGs in the ED dataset were significantly enriched in the ferroptosis, NOTCH pathway, WNT signaling pathway and pluripotency, hippo signaling pathway, fceri-mediated MAPK activation, and HEDGEHOG-, and JAK-STAT -signaling pathways (Supplementary Figures S3A–H). GSVA was conducted to explore the differences in the hallmark gene sets during differentiation. The results showed that 21 hallmark gene sets differed between the long and short groups, such as oxidative phosphorylation, hypoxia, cholesterol homeostasis, WNT/beta-catenin signaling, JAK-STAT3 signaling, PI3K-AKT-MTOR signaling, MTORC1 signaling pathways (*p* < 0.05) (Figure 4A). A grouping comparison map was drawn for these 21 hallmark gene sets (Figure 4B) to further illustrate the expression differences. Differences in the 20 hallmark gene sets were statistically significant between the long and short groups (*p* < 0.05), except for the hallmark early estrogen response gene sets (*p* < 0.05).

### 3.3 Protein-protein interaction network (PPI) construction of ferroptosis related genes

**FIGURE 5:**
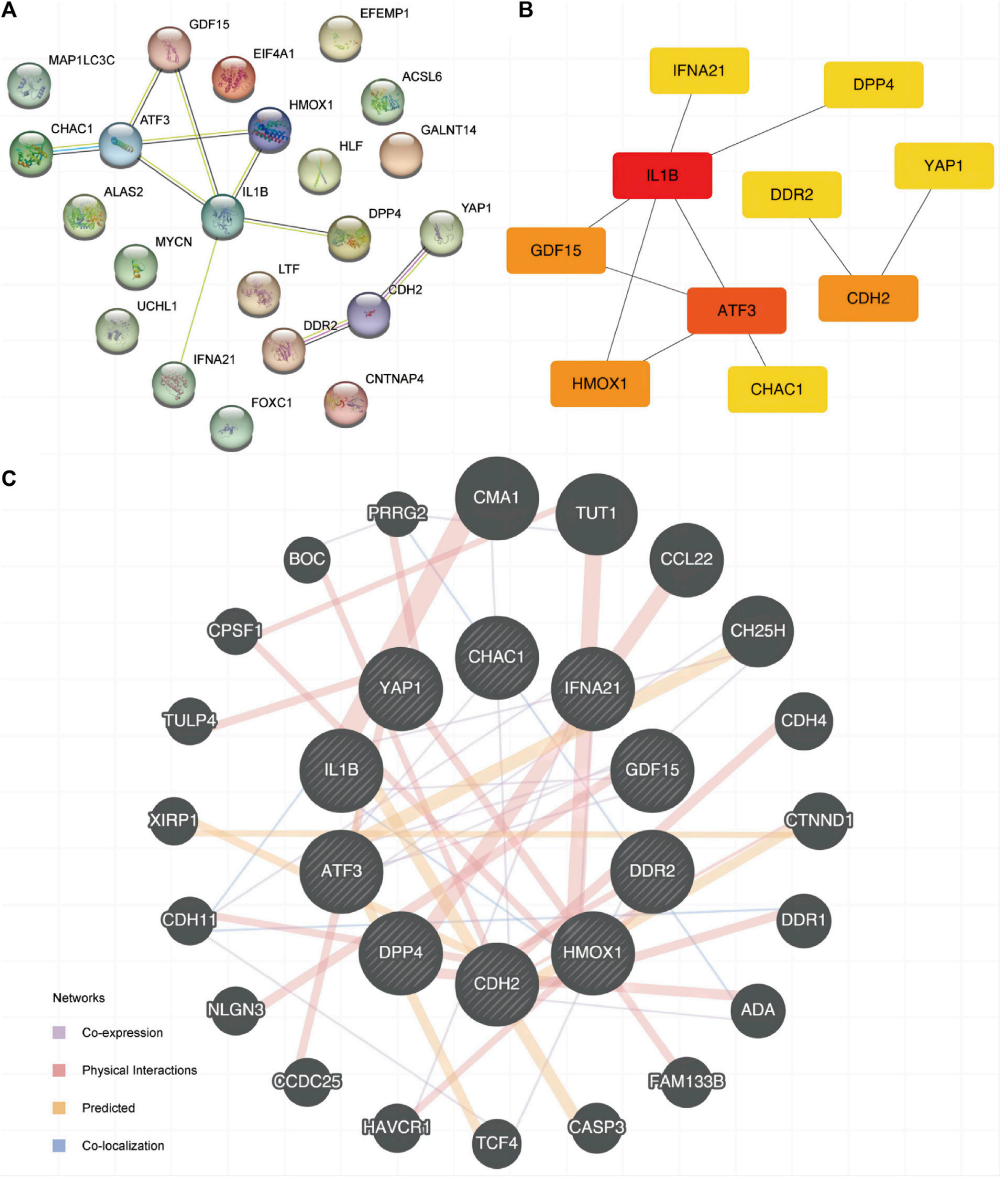
PPI interaction network. **(A)** PPI network of FRGs. **(B)** PPI network of FRGs in MCC algorithm. The rectangle color from yellow to red in the Figure represents the gradual increase of the score. **(C)** FRGs’ GeneMANIA site predicts interaction networks of functionally similar genes. FRGs, ferroptosis-related genes. PPI, protein-protein interaction. MCC, maximal clique centrality.

PPI analysis was conducted to determine the interactive relationship among DE-FRGs (Figure 5A). The cytoHubba plugin analysis identified 10 hub genes (*ATF3, CDH2, CHAC1, DDR2, DPP4, GDF15, HMOX1, IFNA21, IL1B,* and *YAP1*). *IL1B* had the greatest interaction with other FRGs when the least required interaction score was 0.400. Subsequently, we used the maximal clique centrality (MCC) algorithm to calculate the scores of DE-FRGs connected to other PPI network nodes (Figure 5B). Specific FRG scores are listed in Supplementary Table S2. IL1B ranked first in the MCC algorithm score. Additionally, we predicted and constructed an interaction network of functionally similar genes of these 10 DE-FRGs using the GeneMANIA website (Figure 5C) to observe co-expression, physical interaction relationships, prediction, and co-localization. Moreover, the Spearman algorithm was used to analyze the correlation between the 10 FRG expression levels, and the correlation heat map (Supplementary Figure S4A) and chord maps (Supplementary Figure S4B) were used to display the results of the analysis. The results showed that, except for gene DPP4 in the ED dataset, the correlation between the expression levels of *IL1B* and other FRGs was mainly negative, conversely, the correlation between the expression levels of other FRGs was mainly positive. A correlation scatter diagram shows the results of the correlation analysis for the most representative four pairs of genes (Supplementary Figures S3C–F).

**FIGURE 6:**
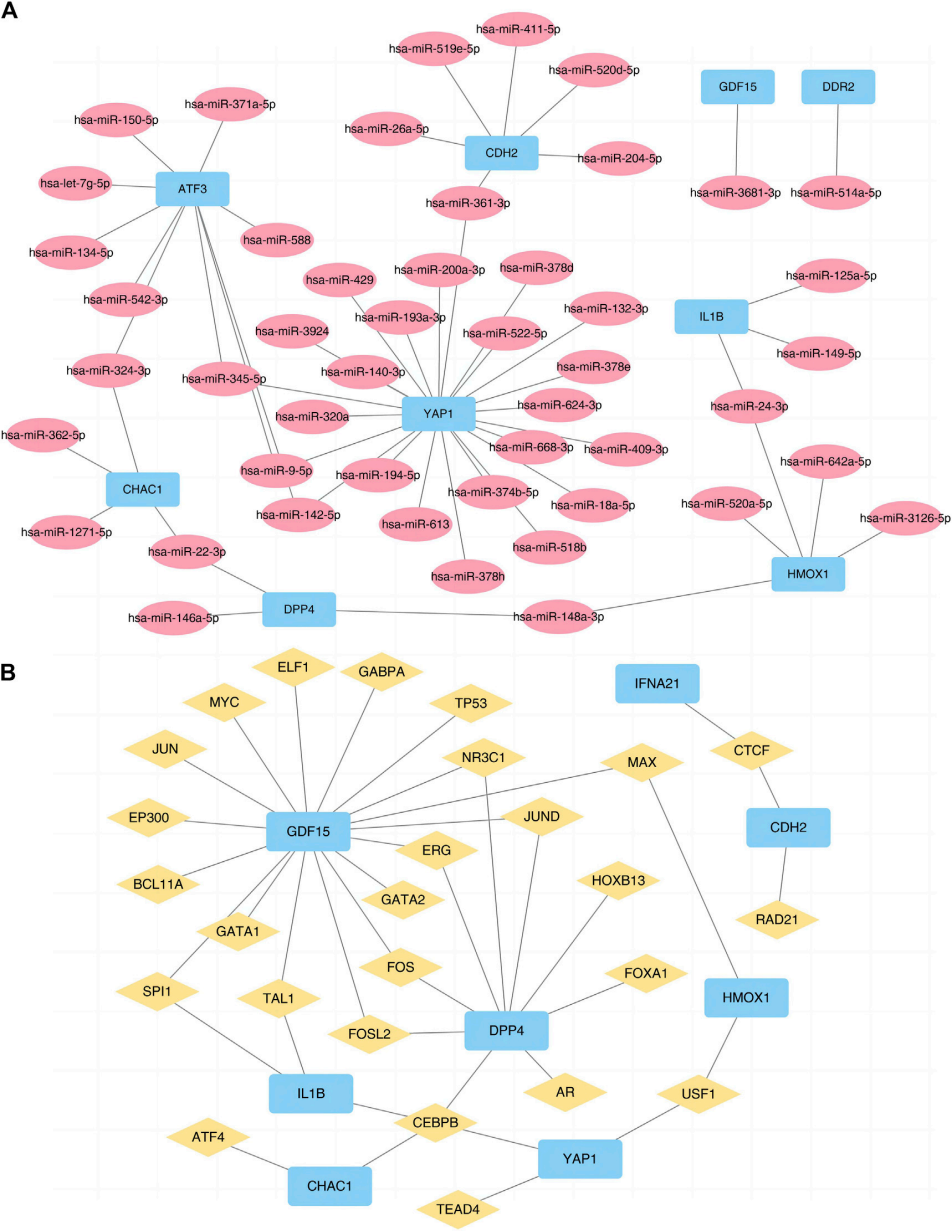
Prediction networks of mRNA-miRNA and mRNA-TF. **(A)** Interactions of DE-FRGs and miRNA. **(B)** Interactions of DE-FRGs and transcription factor. TF, Transcription factor. DE-FRGs, differentially expressed ferroptosis-related genes.

We also used the ENCORI and ChIPBase3.0 database to predict the microRNAs (miRNAs) and TF interacting with the 10 FRGs (Figure 6) to accurately investigate the molecular mechanism underlying the 10 hub DE-FDEGs, and then mapped the interaction network using Cytoscape software. The mRNA-miRNA interaction network consisted of nine mRNAs and 48 miRNAs (Supplementary Table S3). The mRNA-TF interaction network contained eight mRNAs (*CDH2, CHAC1, DPP4, GDF15, HMOX1, IFNA21, IL1B,* and *YAP1*) and 26 TF (Supplementary Table S4).

### 3.4 The subtypes of ED were constructed through consistent clustering

Unsupervised consensus clustering was performed based on DE-FRGs. By increasing the clustering variable k from 2 to 8, we found that optimal categorization occurred when k = 3 (Supplementary Figure S5A–C). Additionally, the principal component analysis (Supplementary Figure S5D) and heat maps (Supplementary Figure S5E) revealed significant differences among the three clusters.

### 3.5 FRGs scores determined using the by ssGSEA algorithm

Based on the expression of the 10 hub DE-FRGs, the ssGSEA algorithm was used to obtain the FRG scores of each sample to represent the FRG expression level. Subsequently, the dataset was then classified into high and low FRG scores, based on the median FRG score. Next, a grouping comparison diagram showed that the FRG scores of the 10 DE-FRGs were significantly different between the high and low groups (Supplementary Figure S6A). The ROC curves for the 10 FRGs (Supplementary Figures S6B–K) showed that the expression of *GDF15* (AUC = 0.938) and *HMOX1* (AUC = 0.981) was highly accurate in predicting FRG scores.

### 3.6 Immune cell infiltration estimated DE-FRGs regulate the immune cell infiltration pattern during ED

**FIGURE 7:**
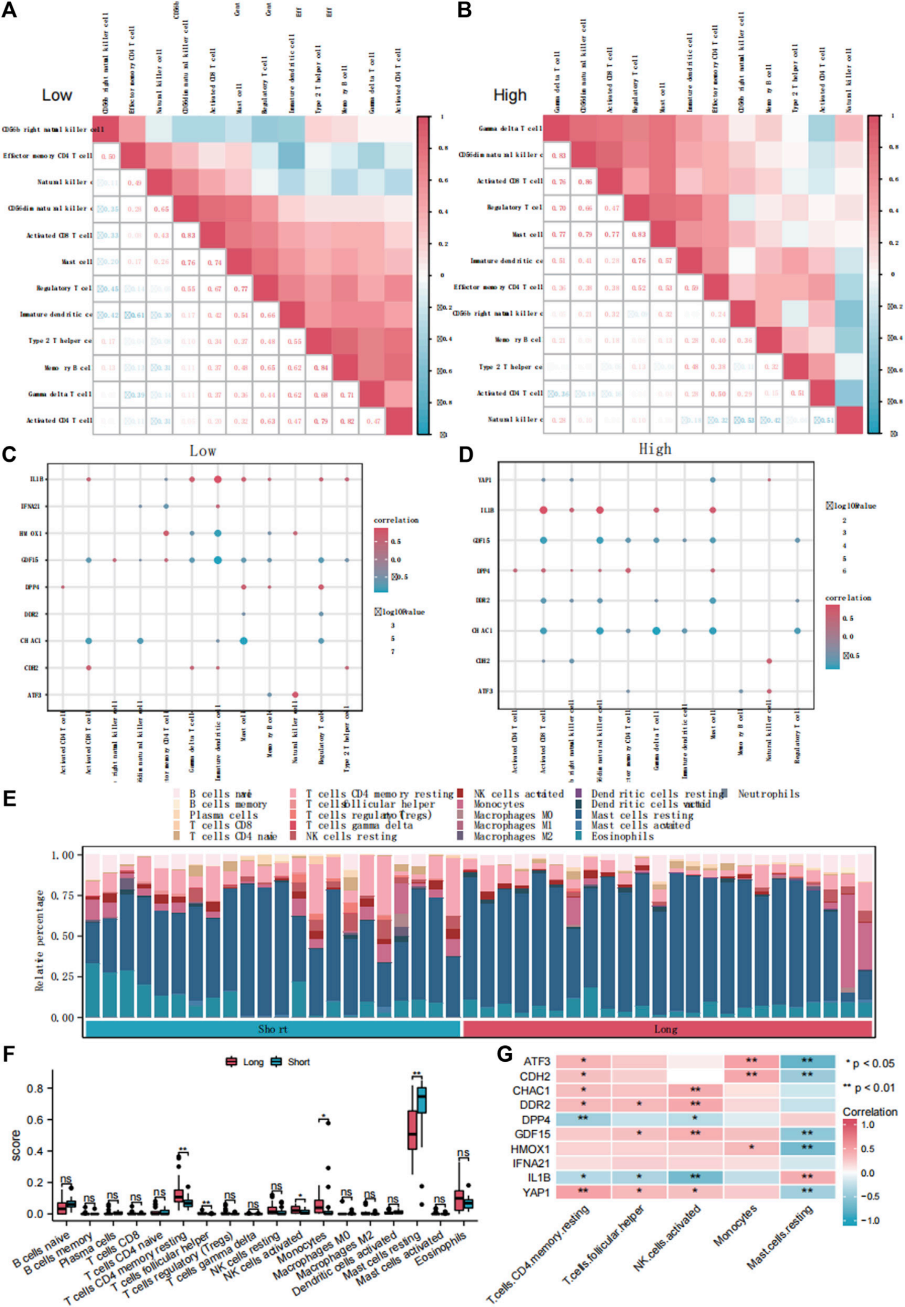
The immune infiltration analysis of ssGSEA and CIBERSORTx. Correlation analysis of immune cell infiltration abundance in dataset ED-dataset with low **(A)** and high **(B)** FRG score. Correlation analysis of immune cells and DE-FRGs in groups with low **(C)** and high **(D)** FRGs score. **(E)** Bars plot showed 22 types of immune cells in different samples of the ED-dataset. **(F)** Box plot showed difference of infiltrating immune cells between the long and short groups based on CIBERSORT. **(G)** Heatmap of correlations of hub genes with differentially infiltrated inED. ED, erythrocyte differentiation. FRGs, ferroptosis-related genes. ssGSEA, single sample gene set enrichment analysis. DE-FRGs, differentially expressed ferroptosis-related genes.

To explore the differences in immune infiltration between the groups with high and low FRG scores, we used the ssGSEA algorithm to calculate the infiltration abundance of 28 immune cell types (Supplementary Figure S7A). The results showed that the infiltration abundances of the 12 immune cell types were significantly different between the high and low FRGs score groups (*p* < 0.05). Subsequently, we then calculated the correlation between the infiltration abundance of these 12 immune cell types in the low (Figure 7A) and high (Figure 7B) FRG score groups. The infiltration abundance of the 12 immune cell types showed a positive correlation (r > 0). Simultaneously, we calculated the abundance of the 12 immune cell types of infiltration and 10 DE-FRGs in the low (Figure 7C) and high (Figure 7D) FRG score groups. The results showed that IL1B was significantly positively correlated with these 12 immune cell types (r > 0, *p* < 0.05), whereas GDF15 and CHAC1 were negatively correlated with the 12 immune cell types (r < 0, *p* < 0.05).

Comparing the immune infiltration of the long- and short groups with CIBERSORTx, significant differences were found in the immune infiltration components between the two groups (Figures 7E, F). In the long group, the proportions of CD4 memory resting T cells, follicular helper T cells, activated NK cells, and monocytes were significantly higher, and resting mast cells were lower than those in the short group (*p* < 0.05) (Figure 7F). The heat map (Figure 7G) shows the correlation between the abundance of the five immune cell infiltrates and the 10 DE-FRGs. Four representative pairs of FRGs and immune cells were collected to construct correlated scatter graphs (Supplementary Figures S7B–E).

### 3.7 LASSO diagnostic and logistic models revealed that DE-FRGs predict differentiation

**FIGURE 8:**
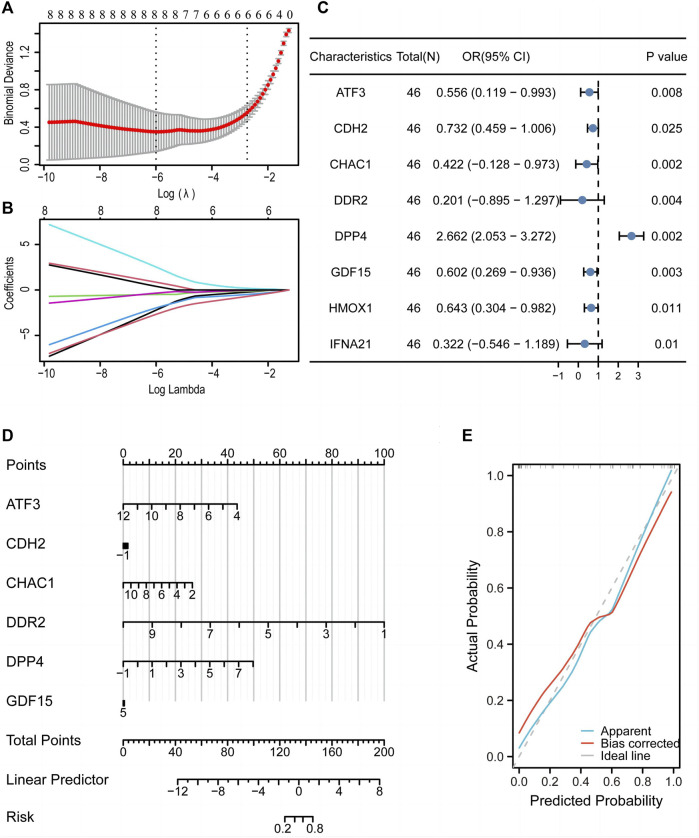
A diagnostic model of the training set was constructed using the LASSO diagnostic model and logistic model. LASSO regression analysis **(A)** and LASSO coefficient **(B)** revealed eight genes associated with diagnosis. **(C)** Forest map of single factor logistic model. **(D)** Nomogram to predict the diagnostic value of the multi-factor Logistic model. **(E)** Diagnostic calibration curve of multi-factor logistic model. LASSO, least absolute shrinkage and selection operator.

LASSO regression analysis was used to construct a diagnostic model (Figure 8A), which revealed eight FRGs for predicting differentiation. We also obtained a trajectory diagram for the LASSO variables (Figure 8B). Subsequently, a logistic model was constructed for the ED dataset with these eight FRGs, and a forest map (Figure 8C) was used to show the situation of the single-factor logistic model. Six FRGs (*ATF3, CDH2, CHAC1, DDR2, DPP4,* and *GDF15*) were included in the multi-factor multiple logistic model. We constructed a diagnostic nomogram (Figure 8D) and a diagnostic calibration curve (Figure 8E). The red line representing the calibration curve is close to the gray line representing the ideal curve, indicating that the model is well-fitted.

### 3.8 Validation of hub genes using *RT-qPCR*

**FIGURE 9:**
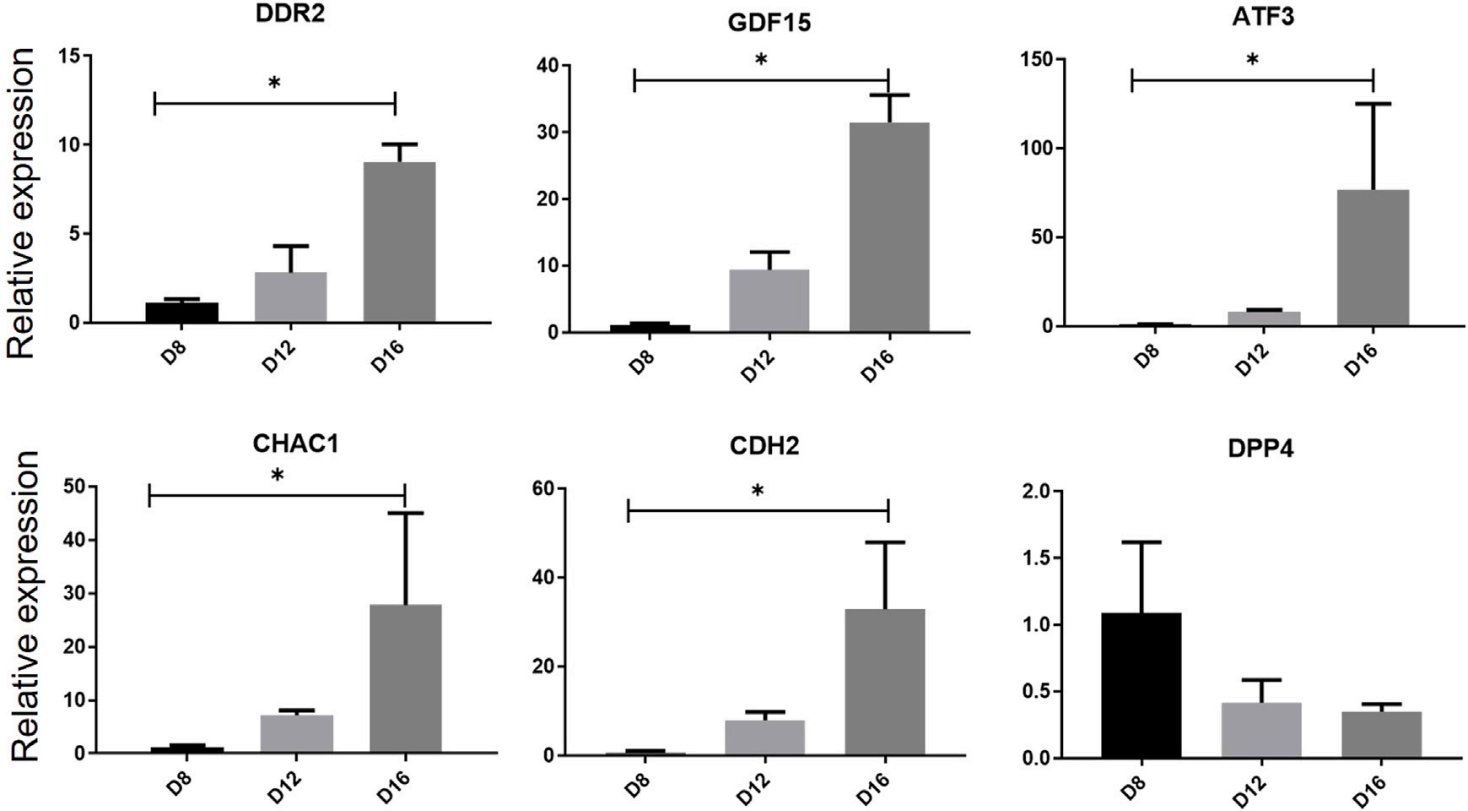
Relative gene expression verification of hub genes during erythrocyte differentiation. The expression levels of hub genes in human umbilical cord blood-derived CD34^+^ on differentiation days 8, 12 and 16 was measured by RT-qPCR. One-way analysis of variance followed by Dunnett’s multiple comparison test was used to compare means among groups. ^*^ *p* < 0.05, ^**^ *p* < 0.01, ^***^ *p* < 0.001. Data are expressed as the mean ± SD of technical triplicates from one of several independent experiments. RT-qPCR, quantitative reverse-transcription PCR.

We previous previously established a serum‐ and feeder‐free culture system to induce and monitor the erythroid differentiation of umbilical cord blood ‐derived CD34^+^ cells. The mRNA expression levels of the six genes in the multifactor multiple logistic model were further evaluated during *in vitro* erythropoiesis (Figure 9). RT-qPCR results showed that the mRNA expression levels of *DDR2, GDF15, CDH2, CHAC1*and ATF3 were upregulated on day 16 compared with that on day 8 (*p* < 0.05), whereas the expression level of DDP4 was downregulated on day 16 (*p* < 0.05) during erythroid differentiation of human umbilical cord blood-derived CD34^+^ cells, consistent with the trend of bioinformatic analysis.

## 4 Discussion

Anemia is a worldwide health issue and a common complication of many diseases, such as heart failure and tumors. Transfusion is a crucial treatment. However, blood supply shortages and transfusion-related risks have become global issues of major concern due to donor-related issues. Despite the substantial efforts made *in vitro* generation of erythrocytes using various stem cells, most protocols have low efficiency in generating sufficient functional red blood cells (RBCs), including stable β-globin expression and enucleation. Therefore, understanding the erythropoiesis mechanism during development is necessary. Erythrocytes, organelle-free cells packaged with iron-containing hemoglobin, are the end-product of a complex hierarchy of hematopoietic progenitors that become progressively restricted to the erythroid lineage. This stepwise differentiation process requires large amounts of iron for hemoglobin synthesis. Ferroptosis is involved in various physiological and pathological processes, including erythropoiesis.

In this study, we explored 753 genes involved in ferroptosis and identified 25 DE-FRGs between the early and late ED stages. Enrichment analysis indicated that DE-FRGs were primarily involved in the JAK-STAT, MAPK, PI3K-AKT-mTORC1, WNT, and NOTCH signaling pathways. Ten hub DE-FRGs were obtained based on the PPI analysis. Furthermore, we created mRNA-miRNA and mRNA-TF constructs. The infiltration levels of immune cells differed significantly during ED. Finally, we established a signature using six DE-FRGs related to ED stages through LASSO regression analysis. The RT-qPCR validation results suggested that the six DE-FRGs are potential signature genes for erythropoiesis. This study revealed that ferroptosis may provide a promising strategy for optimizing *in vitro* erythrocyte hematopoiesis and treating erythrocyte diseases.

This study identified the molecular signatures of ferroptosis in ED of human umbilical cord blood-derived CD34^+^ cells. We obtained 10 hub DE-FRGs with significant interactions (*IL1B, HMOX1, DPP4, ATF3, YAP1, CDH2, CHAC1, DDR2, GDF15*, and *IFNA21*). Similar to the results of our study, *IL1B, HMOX1, GDF15, YAP1*, and *DPP4* have been reported to play roles in ferroptosis and erythroid differentiation. *IL1B* interacted with five FRGs. IL-1β might promote hypoferremia or decreased iron availability by stimulating the expression of hepcidin, which could impair erythropoiesis (Nemeth et al., 2004). IL-1 receptor antagonists reduced the incidence of anemia (Dinarello, 2005; Vallurupalli et al., 2020). Therefore, IL-1β may be decreased to increase iron availability and promote erythroid differentiation. Autophagy inhibits excess iron-induced ferroptosis and subsequently increases IL-1Β (Su et al., 2021). *HMOX1*, the inducible enzyme that catabolizes the degradation of heme into iron, plays a major role in the clearance of senescent and damaged RBCs, systemic iron homeostasis, and erythropoiesis (Starzyński et al., 2013; Kim et al., 2018). The discrepancy in sensitivity to ferroptosis can be determined and regulated by HO-1 (Li et al., 2023). The oxidative stress also promoted HO-1 translocation to mitochondria, leading to mitochondrial iron overload (Chen Y. et al., 2023). GDF-15, a bone marrow-derived cytokine, suppresses the iron regulator hepcidin *in vitro* and is significantly increased in patients with ineffective erythropoiesis (Liu et al., 2021). GDF-15 is the mitochondrial metabolism related markers. Those with iron overload had higher GDF15 levels compared with non-iron overload patients (Huang et al., 2019). *YAP1*, an erythroid regulator, coordinates metabolic status with the proliferation of erythroid progenitors to promote stress erythropoiesis (Hao et al., 2019). YAP/TAZ play an important role in erythroid maturation and enucleation (Damkham et al., 2022). Hippo/YAP1/c-Jun axis regulated iron metabolism (Zhou et al., 2023). YAP1 deficiency boosted mitochondrial dysfunction and the ferrous iron accumulation (Zhang J. et al., 2022). DPP-4 decreases erythropoietin (Epo) activity by cleavage, negatively regulates colony-stimulating factor activity and stress hematopoiesis (Broxmeyer et al., 2012; Ou et al., 2013), and improves the responsiveness to erythropoiesis-stimulating agent (Hasegawa et al., 2021). In our study, we predicted the differential expression of these genes in the early and late stages of differentiation and analyzed the pathways and immune infiltration associated with these genes in subsequent analyses. The transcription factors ATF3 and YAP1 play multiple roles in shaping ferroptosis sensitivity through either transcription-dependent or transcription-independent mechanisms (Dai et al., 2020). As there is still a lack of evidence on whether FRGs contribute to the stage of ED, a logistic regression model was constructed to distinguish between five key genes (*DPP4, CDH2, CHAC1,* and *DDR2*). The validation of the six key genes met the trend of bioinformatic analysis. However, a few studies have investigated the mechanisms of action of other genes involved in ED.

Furthermore, functional enrichment analysis indicated that the DEGs were primarily involved in the JAK-STAT, MAPK, PI3K-AKT-mTORC1, WNT, and NOTCH signaling pathways. Hypoxia promotes erythropoiesis by increasing Epo production. Although Epo is the principal regulator of erythroid progenitors, signaling from the Epo-receptor activates several pathways, including the JAK/STAT, ras/raf/MAP kinase, and PI3K/Akt cascades, to promote cell survival, proliferation, and differentiation (Ingley et al., 2004). JAK/STAT, MAPK, and PI3K are considered the main signaling pathways that play significant roles in fetal hemoglobin induction (Rahim et al., 2013). The JAK-STAT signaling pathway regulates certain TFs (GATA1, GATA2, SPI1, and RUNX1) involved in ED regulation, development, and maturation (Liu et al., 2017). Erythrocyte proliferation and survival are also associated with the activation of the JAK-STAT pathway (Cokic et al., 2012). The level of IL-1β was decreased via inactivating of JAK/STAT signaling pathway (Ni et al., 2023). The PI3K-AKT signaling pathway regulates the Epo-induced survival, proliferation, and maturation of early erythroid progenitors (Lopez et al., 2011). The reduction of Pi4ka inhibits myeloid and erythroid cell differentiation *in vitro* and promotes anemia *in vivo* through a mechanism involving the deregulation of AKT, MAPK, and JAK-STAT signaling pathways (Ziyad et al., 2018). Ferroptosis can be reducd through the MAPK signaling pathway (Chen W. et al., 2023). We previously reported the mTOR signaling pathway played roles during human umbilical cord blood-derived CD34^+^ cell erythropoiesis *in vitro* (Liu et al., 2020). The inhibition of mTORC1 sensitizes cells to ferroptosis (Zhang et al., 2021). The components of the JAK-STAT-NF-κB signaling pathway are DNA hypomethylated and upregulation, targeting key genes for erythropoiesis. The activation of the Wnt/beta-catenin signaling attenuates cellular lipid ROS production and subsequently inhibits ferroptosis. The beta-catenin/TCF4 transcription complex directly binds to the promoter region of GPX4, a peroxidase that suppresses ROS-triggered ferroptosis resulting in suppressing ferroptosis (Wang et al., 2022). Although Wnt/β-catenin is dispensable for steady-state erythropoiesis, its activity is essential for stress erythropoiesis in response to bone marrow injury and anemia (Krimpenfort and Nethe, 2021). The canonical WNT signaling pathway also inhibits the expansion and/or survival of primitive erythrocytes (Paluru et al., 2014). GATA2 contributes to the inhibition of the canonical WNT signaling pathway, thereby permitting progenitors to exit the cell cycle and commit to a hematopoietic fate. Subsequently, the activation of the non-canonical WNT signaling pathway plays a role in enabling progenitors to differentiate into mature RBCs (Mimoto et al., 2015). The activation of the NOTCH signaling pathway leads to the inhibition of differentiation of immature precursors, suggesting important roles for the NOTCH signaling pathway in hematopoiesis (Sugimoto et al., 2006). Our results expand our understanding of the mechanisms underlying erythropoiesis. However, broader validation is needed to improve our understanding of the mechanisms of ferroptosis in the ED of human umbilical cord blood-derived CD34^+^ cells.

Erythropoiesis is a complex and sophisticated multistage process regulated by TFs and miRNAs. This study also established TF- and miRNA-target gene networks. The importance of several TFs in erythropoiesis has been unequivocally demonstrated by cell-based *ex vivo* assays, as well as in knockout mouse models and rare patients with anemias (Kerenyi and Orkin, 2010). Members of the GATA TF family, GATA1 and GATA2, play crucial roles in regulating lineage-restricted genes during erythroid differentiation (Moriguchi and Yamamoto, 2014). GATA-2 is essential for the maintenance and proliferation of immature hematopoietic progenitors, whereas GATA-1 is essential for the survival of erythroid progenitors and terminal differentiation of erythroid cells (Ohneda and Yamamoto, 2002). Similarly, in our differentiation system, GATA1 expression gradually increased and then decreased, whereas GATA2 expression gradually decreased following cell maturation (Liu et al., 2020). Several TFs, such as GATA-1 (Vakoc et al., 2005) or BCL11A (Sankaran et al., 2008), have been described as being required for the transcriptional switch from γ-globin to β-globin expression. BCL11A plays a novel regulatory role in erythroid differentiation, maturation, and globin production (Luanpitpong et al., 2022). MiRNAs play key roles in erythropoiesis and control the expression of several TF genes involved in erythroid differentiation and hemoglobin gene expression (Jafari et al., 2019). MiRNA-146a is significantly more abundant in reticulocytes obtained from adults than those from umbilical cord blood and inhibits γ globin expression (Azzouzi et al., 2011). MiR‐24 promotes terminal differentiation (Drak Alsibai and Meseure, 2018). Our findings provide a resource for future studies aimed at elucidating the roles of TFs and miRNAs in erythropoiesis.

The blood and immune systems develop during early embryogenesis. Immune-erythroid cells are coupled with dual erythroid and immune regulatory networks and play immunomodulatory roles throughout human ontogenesis by actively interacting with various immune cells (Xu et al., 2022). Two transcriptional regulation programs are co-activated in immune erythroid cells: one is centered on the GATA1, MYC, and MYB regulons to ensure normal erythroid differentiation, and the other is dependent on the GATA2, FOS, and JUN regulons to instruct immunomodulatory activity. The mRNA-TF interaction network in our study included GDF15, GATA1, MYC, MYB, GATA2, FOS, and JUN. A negative correlation was found between GDF15 and the abundance of the 12 immune cell types of infiltration in samples with high and low FRG scores. GDF15 was associated with the abundance of five immune cell types between the early and late differentiation stages. Therefore, we speculated that GDF15 is an important gene associated with ED, ferroptosis, and immune invasion. Our results showed that some immune cells differed significantly during erythropoiesis. Erythroid progenitor cells play an important role in the regulation of immune responses and tumor progression. CD71+-nucleated erythroid cells play immunosuppressive roles in orchestrating immune response (Grzywa et al., 2021). RBCs affect immune cell functions. For example, they inhibit T-cell proliferation via direct cell-cell contact and affect dendritic cell functions (Bernard et al., 2010; Yi et al., 2015). Moreover, RBCs serve as critical immune sensors through the surface expression of nucleic acid–sensing toll-like receptor 9 (Lam et al., 2021). Endothelial progenitor cell (EPC) differentiation is a promising strategy to reduce cancer-induced immunosuppression and tumor-promoting effects of EPCs (Zhang H. et al., 2022).

Ferroptosis is associated with the immune response. ssGSEA showed that the infiltration abundance of 12 immune cell types was significantly different between high and low FRG scores. Ferroptosis affects immune cells in two ways. Immune cells require sufficient amounts of iron for their proliferation and for mediating their effector function. Ferroptosis affects the number and functions of immune cells. In contrast, ferroptotic cells are recognized by immune cells and trigger various inflammatory or specific responses (Chen et al., 2021). Ferroptosis regulates the activity and function of cytotoxic (CD8^+^) and helper T (CD4^+^) cells. Ferroptosis mediates neutrophil recruitment and neutrophil extracellular trap formation (NETosis). In T cells, ferroptosis induces a novel synergy between immunotherapy and radiotherapy. Additionally, ferroptosis may mediate B cell differentiation, antibody responses, and lymphoma. Ferroptosis regulates T- and B-cell immunity, which are involved in infectious diseases. Chronic immune activation in the setting of malignancy alters systemic iron homeostasis and directs iron fluxes into myeloid cells, which may result in iron-restricted erythropoiesis and anemia (Pfeifhofer-Obermair et al., 2018). Tumor-induced erythroid progenitor cells eventually differentiate into myeloid cells that exert robust immunosuppressive functions (Long et al., 2022). Consistent with our results, an interaction was found between the immune status, ferroptosis, and erythropoiesis. However, the exact mechanisms underlying these interactions require further study.

Considering the individual differences and technical limitations, we integrated three gene expression profile datasets to obtain a larger sample size and improve the accuracy of our conclusions. However, the number of patients included in this study was relatively small. Another apparent limitation was that we did not perform *in vitro* or *in vivo* experiments to validate the underlying mechanisms and their correlation between immune cells and hub genes, although we verified that six DE-FRGs changed markedly during ED stages. Therefore, we will continue to improve this aspect in the future.

## 5 Conclusion

In our study, 25 hub DE-FRGs were identified for erythropoiesis, which could be potential targets for subsequent research. Functional annotations were performed to elucidate the processes and pathways involving these genes. Ten hub DE-FRGs were obtained according to the PPI analysis. This study also predicted certain target miRNAs and TF that may be related to the pathophysiological process of erythropoiesis via ferroptosis. In addition, our analysis confirmed differences in immune infiltration during erythropoiesis. Finally, we established a signature using six DE-FRGs related to erythrocyte differentiation stages by LASSO regression analysis. The six DE-FRG were validated to be potential signature genes for erythropoiesis. Our findings may lay the groundwork for future clinical applications of *ex vivo* production of functional human reticulocytes for transfusion of human umbilical cord blood-derived CD34^+^ cells. This study could be important for understanding erythropoiesis and hematologic disorders whose etiology is related to impaired erythroid differentiation and hemoglobinopathies.

## Data Availability

The datasets presented in this study can be found in online repositories. The names of the repository/repositories and accession number(s) can be found in the article/Supplementary Material.

## Supplementary material

SUPPLEMENTARY FIGURE S1 Data preprocessing. **(A)** Box plot of the sample distribution of ED-dataset before normalization. **(B)** Box plot of the sample distribution of ED-dataset after normalization. ED, erythrocyte differentiation.

SUPPLEMENTARY FIGURE S2 A diagnostic assessment of 22 DE-FRGs in the ED-dataset was displayed. A ROC curve was generated to assess the model performance. The AUC is used to determine the prediction accuracy. The AUC is usually between 0.5 and 1.0. The ROC curve has low/moderate/high accuracy when the AUC is 0.5–0.7/0.7–0.9/>0.9, respectively. FRGs, ferroptosis-related genes. ROC, receiver operating characteristic curve. AUC, area under the curve. TPR, true positive rate. FPR, false positive rate. CI, confidence interval. ED, erythrocyte differentiation.

SUPPLEMENTARY FIGURE S3 Correlation analysis of DE-FRGs in ED-dataset. **(A)** The heat map showed the correlation of DE-FRGs. **(B)** The chord map shows the correlation of DE-FRGs. **(C–F)** The scatter plot shows the correlation between *ATF3* and *HMOX1*, *DDR2* and *GDF15, GDF15* and *IL1B,* andDDR2 and IL1B. Correlations were calculated using Spearman correlation analysis. The correlation coefficient (r) in the correlation scatter plot is strongly correlated when the absolute value is above 0.8. The absolute value of 0.5–0.8 is moderately correlated. The absolute value between 0.3 and 0.5 shows a weak correlation. Absolute values below 0.3 are considered weak or irrelevant. FRGs, ferroptosis-related genes. ED, erythrocyte differentiation.

SUPPLEMENTARY FIGURE S4 GSEA. **(A)** Seven main biological characteristics of GSEA of ED-dataset. Genes of ED-dataset were significantly enriched in wp ferroptosis **(B)**, NOTCH pathway **(C)**, WNT signaling pathway and pluripotency **(D)**, HIPPO signaling **(E)**, e fceri mediated MAPK activation **(F)**, hedgehog signaling pathway **(G)**, JAK-STAT signaling pathway **(H)**.

SUPPLEMENTARY FIGURE S5 Consensus clustering analysis based on DE-FRGs **(A)** Consensus heatmap of K = 3. **(B)** Cumulative distribution function (CDF) curves for k = 2–8. **(C)** Elbow plot showing the relative change in area under the CDF curve. **(D)** PCA for the unsupervised clustering results (K = 3). **(E)** The heatmap shows the expression of the 10 hub DE-FRGs among clusters of the dataset. based on the ED-dataset. PCA, principal component analysis. ED, erythrocyte differentiation.

SUPPLEMENTARY FIGURE S6 Construction of FRGs scores **(A)** Grouping comparison graphs of high and low FRGs scores in the ED-dataset are shown. **(B–K)** FRG ROC curve results are displayed in the ED-dataset based on the FRG score group. A ROC curve was generated to assess the model performance. * *P* < 0.05, ** *P* < 0.01, *** *P* < 0.001, ED, erythrocyte differentiation. FRGs, ferroptosis-related genes. ROC, receiver operating characteristic curve. AUC, area under the curve. TPR, true positive rate. FPR: false positive rate. CI, confidence interval.

SUPPLEMENTARY FIGURE S7 The immune infiltration analysis of ssGSEA and CIBERSORTx. **(A)** Grouping comparison of ssGSEA immune infiltration analysis between groups with high and low FRG scores. Correlation analysis of immune cells and DE-FRGs in groups with low FRGs score. Correlation scatter plot of ATF3-Monocytes **(B)**, YAP1 - CD4 memory resting T cells **(C)**, ATF3–Mast cells resting **(D)**, and HMOX1-Mast cells resting **(E)**. Correlations were calculated using Spearman correlation analysis. ED, erythrocyte differentiation. FRGs, ferroptosis related genes. ssGSEA, single sample gene set enrichment analysis.
