# Supplementary material for: Identification and validation of ferroptosis related markers in erythrocyte differentiation of umbilical cord blood-derived CD34+ cell by bioinformatic analysis
Source: Front Genet. 2024 Jul 30;15:1365232. doi: 10.3389/fgene.2024.1365232 (PMC11319168; doi:10.3389/fgene.2024.1365232)
Supplement: Supplementary file 1 [file Table1.DOCX]

Table S1：Real-time polymerase chain reaction primers

| **Primer sequence** | | |
| --- | --- | --- |
| *DPP4* | Forward primer | GAATGCCAGGAGGAAGGAATCT |
|  | Reverse primer | CAGGACCGGAACATCTCAGC |
| *GDF15* | Forward primer | CAGGACGGTGAATGGCTCTC |
|  | Reverse primer | CAGGTCCTCGTAGCGTTTCC |
| *CDH2* | Forward primer | ATGTGCCGGATAGCGGGA |
|  | Reverse primer | TGCGATTTCACCAGAAGCCT |
| *CHAC1* | Forward primer | TGGATTTTCGGGTACGGCTC |
|  | Reverse primer | TTGCTTACCTGCTCCCCTTG |
| *ATF31* | Forward primer | CTCGGGGTGTCCATCACAAA |
|  | Reverse primer | GGCACTCCGTCTTCTCCTTC |
| *DDR21* | Forward primer | GACGATTTCACCCAGACC |
|  | Reverse primer | TCAGAGCGGAAGTAGCAC |
| *ATCB* | Forward primer | TCGTGATGGACTCCGGTGAC |
|  | Reverse primer | TCGTGGATGCCACAGGACTC |

### Table S2. FRGs in PPI network ranked by MCC method.

| Rank | Name | Score |
| --- | --- | --- |
| 1 | IL1B | 6 |
| 2 | ATF3 | 5 |
| 3 | HMOX1 | 2 |
| 3 | CDH2 | 2 |
| 3 | GDF15 | 2 |
| 6 | CHAC1 | 1 |
| 6 | DPP4 | 1 |
| 6 | YAP1 | 1 |
| 6 | IFNA21 | 1 |
| 6 | DDR2 | 1 |

FRGs，ferroptosis related genes. PPI，protein-protein interaction. MCC，maximal clique centrality.

### Table S3. List of interaction relationships of mRNA-miRNA network.

| mRNA | miRNA |
| --- | --- |
| ATF3 | hsa-let-7g-5p |
| ATF3 | hsa-miR-142-5p |
| ATF3 | hsa-miR-9-5p |
| ATF3 | hsa-miR-134-5p |
| ATF3 | hsa-miR-150-5p |
| ATF3 | hsa-miR-324-3p |
| ATF3 | hsa-miR-345-5p |
| ATF3 | hsa-miR-588 |
| ATF3 | hsa-miR-542-3p |
| ATF3 | hsa-miR-371a-5p |
| CDH2 | hsa-miR-26a-5p |
| CDH2 | hsa-miR-204-5p |
| CDH2 | hsa-miR-519e-5p |
| CDH2 | hsa-miR-520d-5p |
| CDH2 | hsa-miR-411-5p |
| CDH2 | hsa-miR-361-3p |
| CHAC1 | hsa-miR-22-3p |
| CHAC1 | hsa-miR-362-5p |
| CHAC1 | hsa-miR-324-3p |
| CHAC1 | hsa-miR-1271-5p |
| DDR2 | hsa-miR-514a-5p |
| DPP4 | hsa-miR-22-3p |
| DPP4 | hsa-miR-148a-3p |
| DPP4 | hsa-miR-146a-5p |
| GDF15 | hsa-miR-3681-3p |
| HMOX1 | hsa-miR-24-3p |
| HMOX1 | hsa-miR-148a-3p |
| HMOX1 | hsa-miR-520a-5p |
| HMOX1 | hsa-miR-642a-5p |
| HMOX1 | hsa-miR-3126-5p |
| IL1B | hsa-miR-24-3p |
| IL1B | hsa-miR-125a-5p |
| IL1B | hsa-miR-149-5p |
| YAP1 | hsa-miR-18a-5p |
| YAP1 | hsa-miR-132-3p |
| YAP1 | hsa-miR-142-5p |
| YAP1 | hsa-miR-9-5p |
| YAP1 | hsa-miR-193a-3p |
| YAP1 | hsa-miR-194-5p |
| YAP1 | hsa-miR-320a |
| YAP1 | hsa-miR-200a-3p |
| YAP1 | hsa-miR-345-5p |
| YAP1 | hsa-miR-429 |
| YAP1 | hsa-miR-409-3p |
| YAP1 | hsa-miR-518b |
| YAP1 | hsa-miR-613 |
| YAP1 | hsa-miR-668-3p |
| YAP1 | hsa-miR-140-3p |
| YAP1 | hsa-miR-361-3p |
| YAP1 | hsa-miR-624-3p |
| YAP1 | hsa-miR-374b-5p |
| YAP1 | hsa-miR-522-5p |
| YAP1 | hsa-miR-3924 |
| YAP1 | hsa-miR-378d |
| YAP1 | hsa-miR-378e |
| YAP1 | hsa-miR-378h |

### Table S4 List of interaction relationships of mRNA-TF network.

| mRNA | TF |
| --- | --- |
| CDH2 | CTCF |
| CDH2 | RAD21 |
| CHAC1 | ATF4 |
| CHAC1 | CEBPB |
| DPP4 | AR |
| DPP4 | CEBPB |
| DPP4 | ERG |
| DPP4 | FOS |
| DPP4 | FOSL2 |
| DPP4 | FOXA1 |
| DPP4 | HOXB13 |
| DPP4 | JUND |
| DPP4 | NR3C1 |
| GDF15 | ELF1 |
| GDF15 | EP300 |
| GDF15 | ERG |
| GDF15 | FOS |
| GDF15 | FOSL2 |
| GDF15 | GABPA |
| GDF15 | GATA1 |
| GDF15 | GATA2 |
| GDF15 | JUN |
| GDF15 | JUND |
| GDF15 | MAX |
| GDF15 | BCL11A |
| GDF15 | MYC |
| GDF15 | NR3C1 |
| GDF15 | SPI1 |
| GDF15 | TAL1 |
| GDF15 | TP53 |
| HMOX1 | MAX |
| HMOX1 | USF1 |
| IFNA21 | CTCF |
| IL1B | SPI1 |
| IL1B | TAL1 |
| IL1B | CEBPB |
| YAP1 | TEAD4 |
| YAP1 | CEBPB |
| YAP1 | USF1 |

TF，Transcription factor.
