# Supplementary figures and images for: Identification and validation of ferroptosis related markers in erythrocyte differentiation of umbilical cord blood-derived CD34+ cell by bioinformatic analysis
Source: Front Genet. 2024 Jul 30;15:1365232. doi: 10.3389/fgene.2024.1365232 (PMC11319168; doi:10.3389/fgene.2024.1365232)

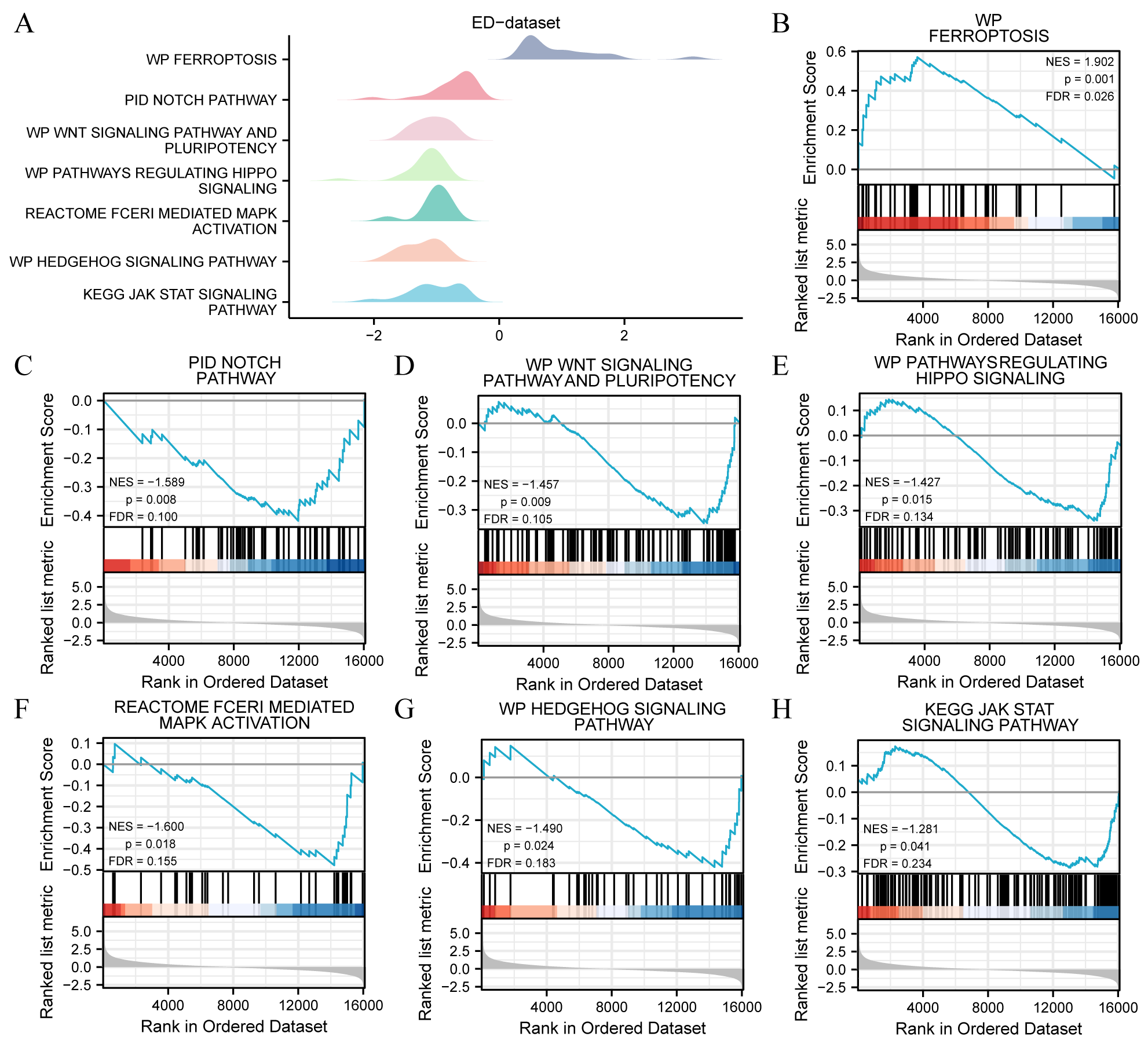

Supplement: Supplementary file 2 [file Image3.TIF]

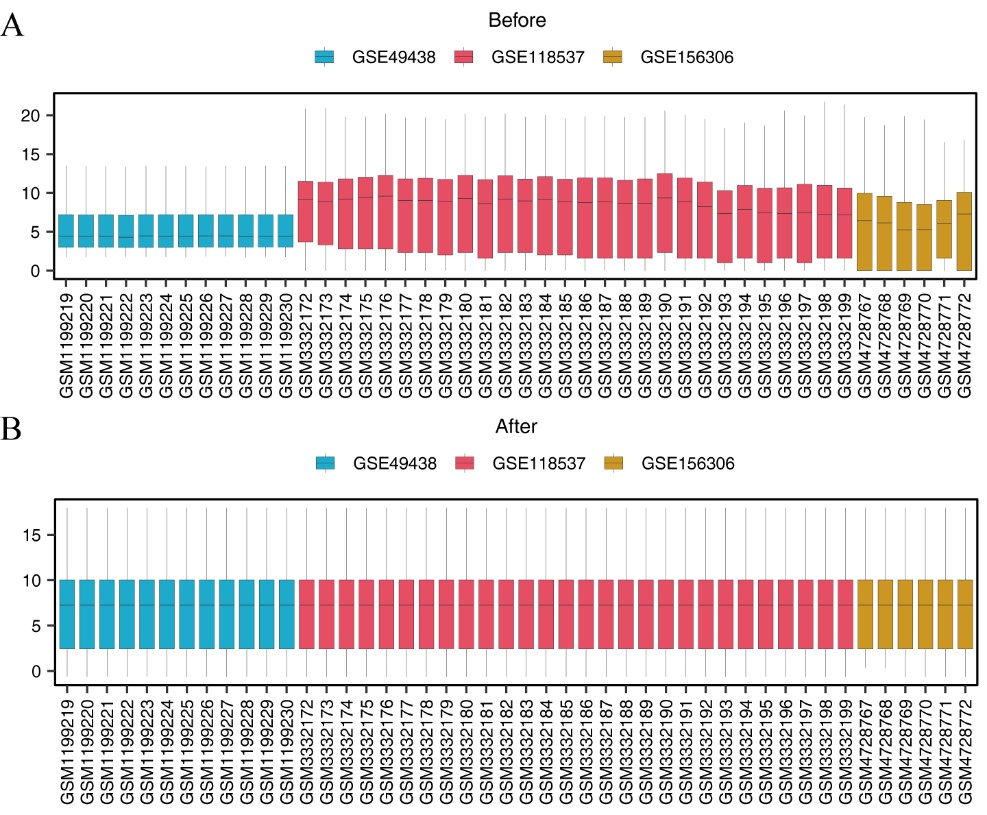

Supplement: Supplementary file 3 [file Image1.JPEG]

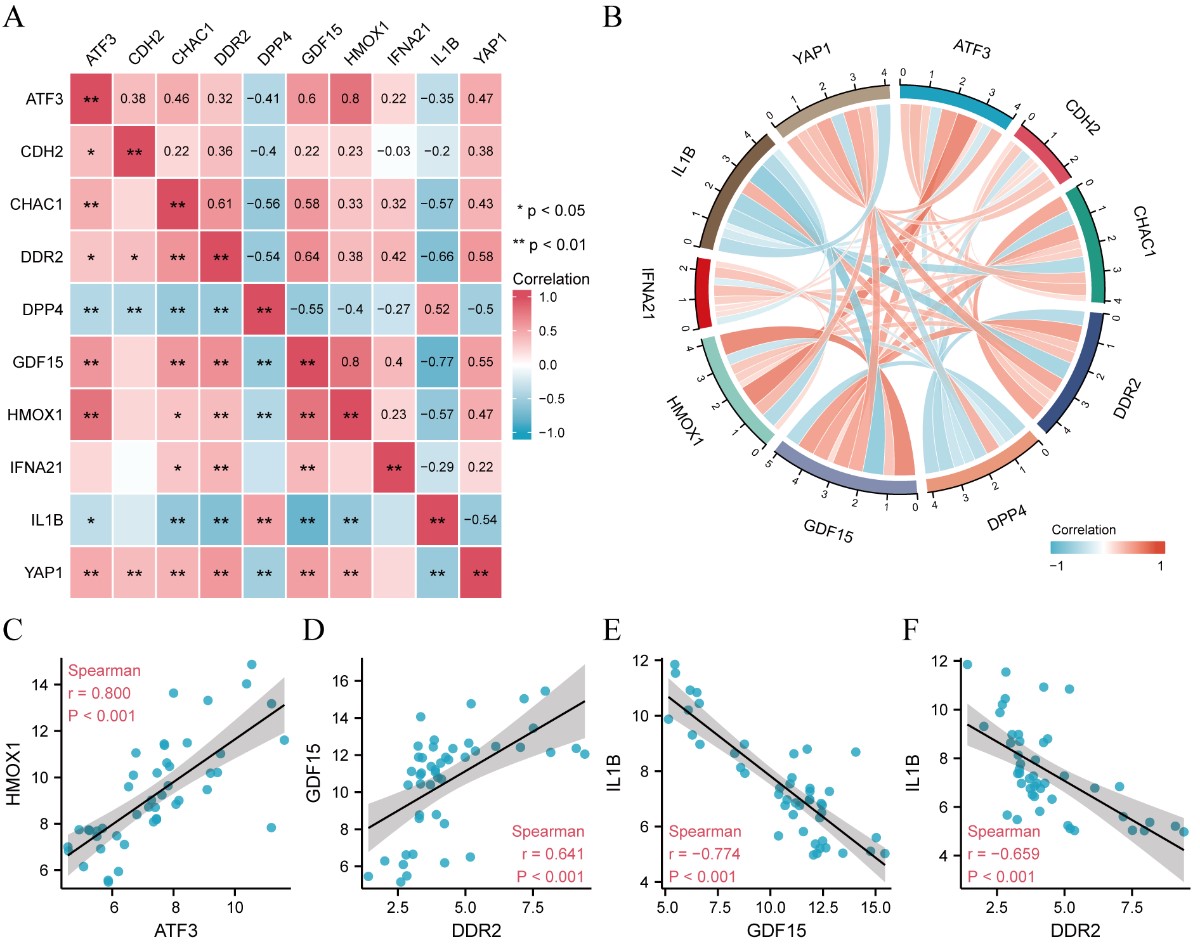

Supplement: Supplementary file 4 [file Image4.JPEG]

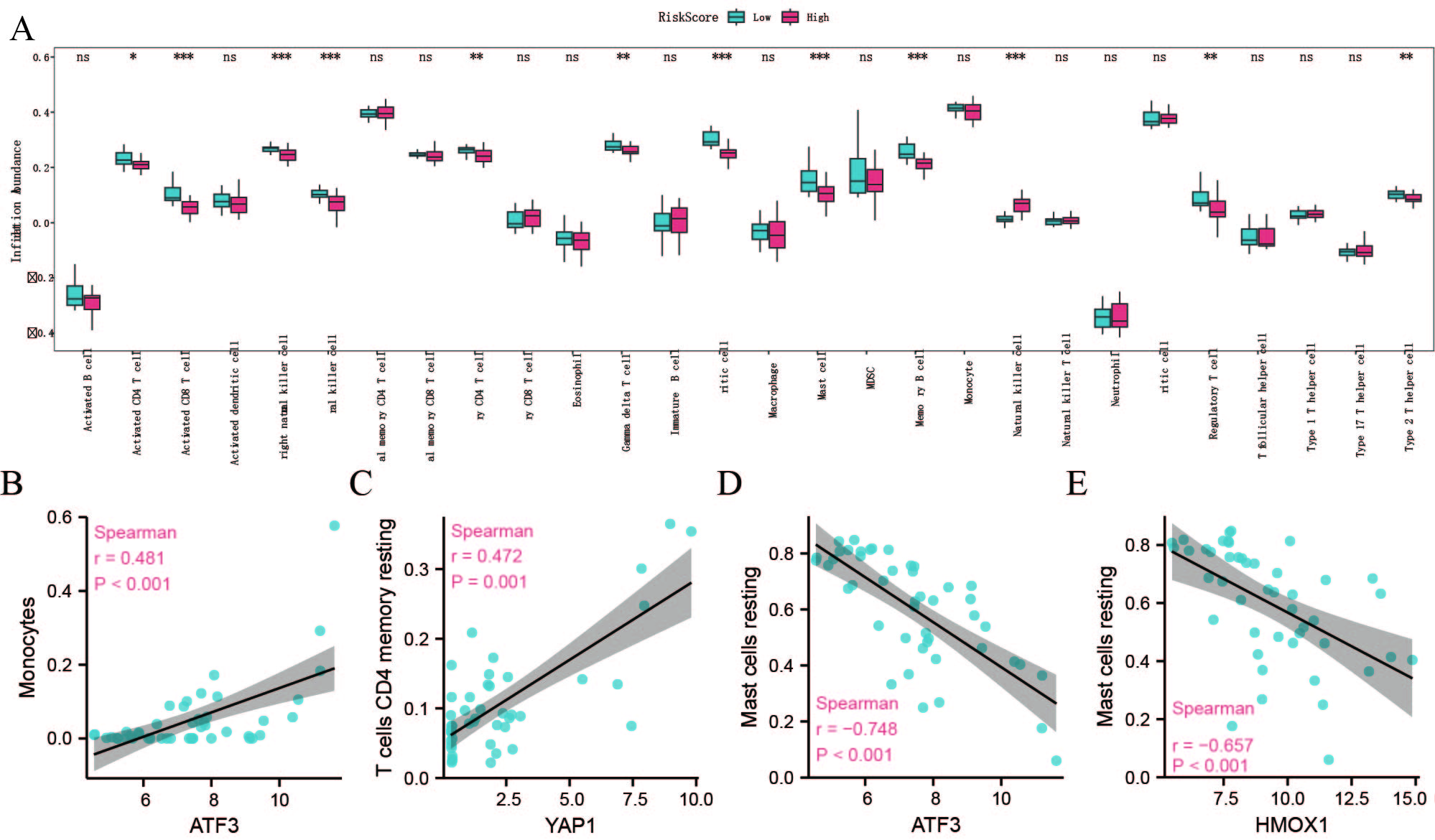

Supplement: Supplementary file 5 [file Image7.JPEG]

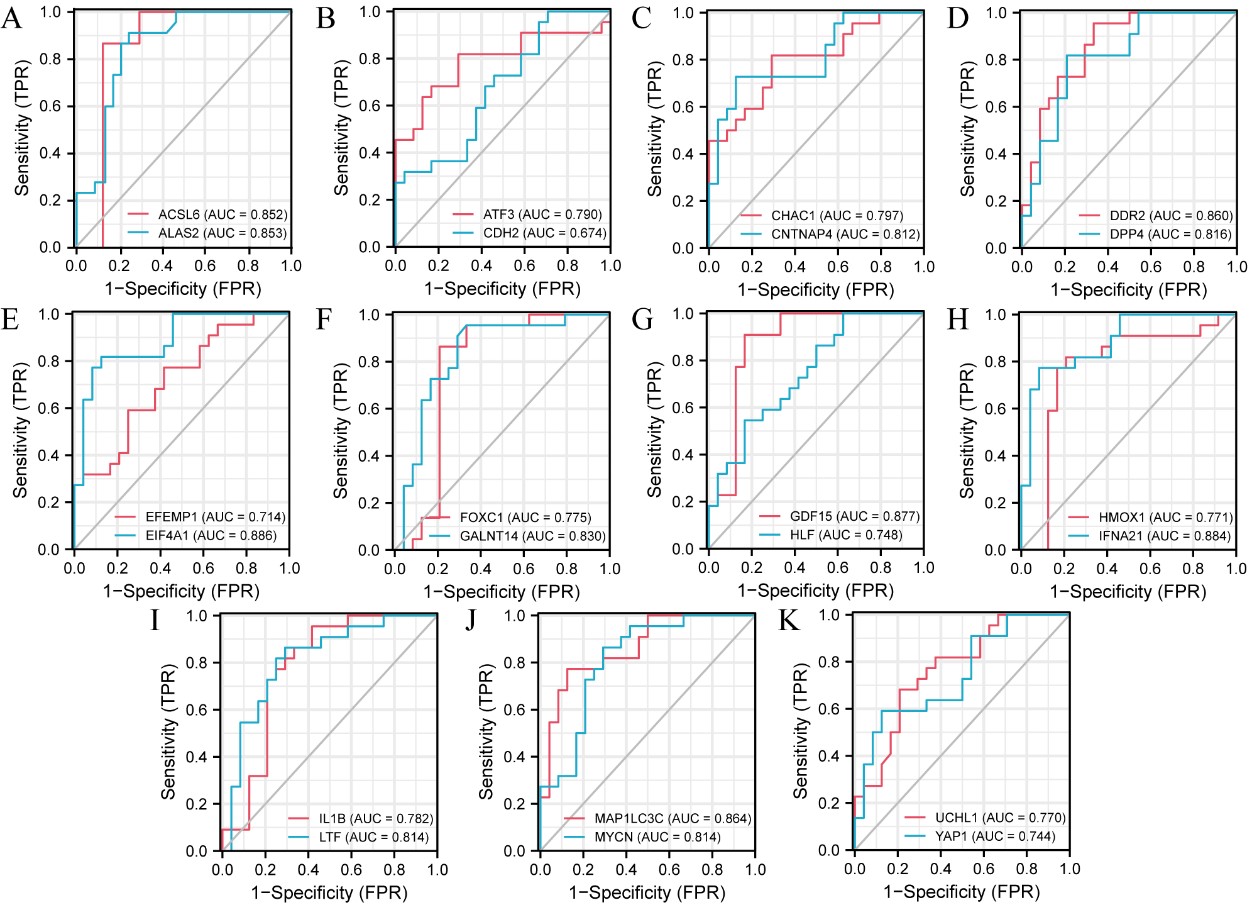

Supplement: Supplementary file 6 [file Image2.JPEG]

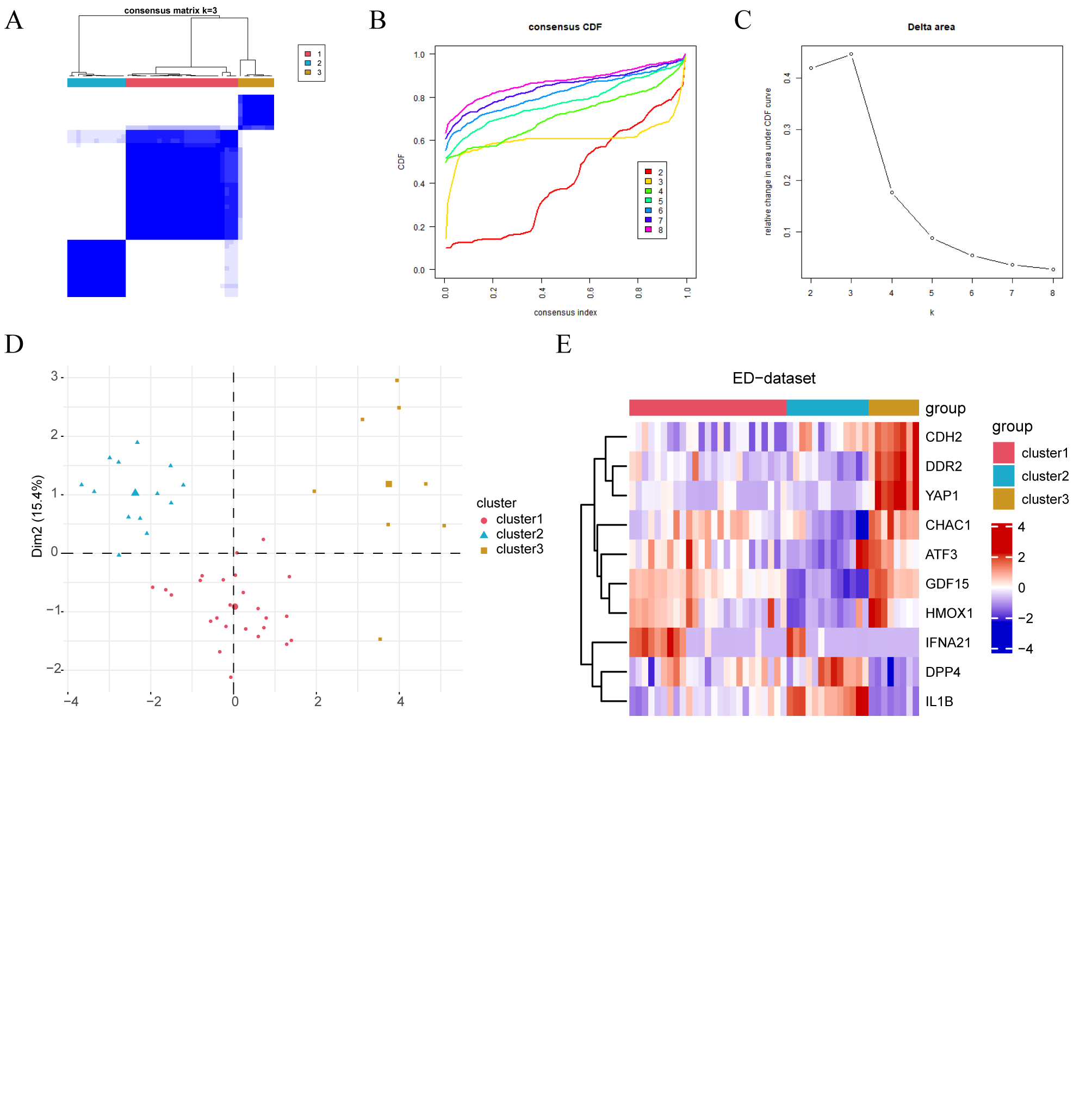

Supplement: Supplementary file 7 [file Image5.TIF]

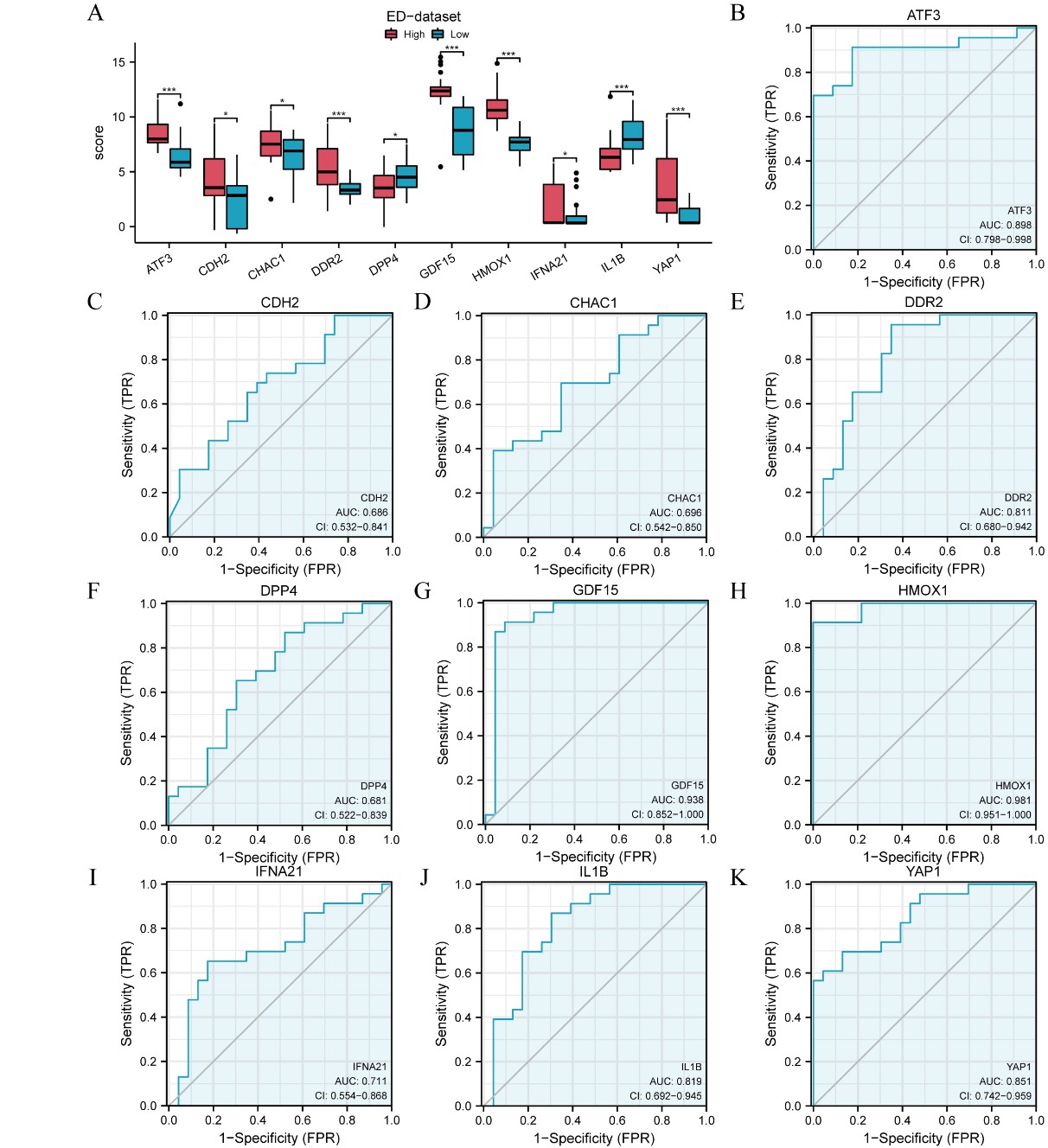

Supplement: Supplementary file 8 [file Image6.JPEG]
